# Targeting aryl hydrocarbon receptor functionally restores tolerogenic dendritic cells derived from patients with multiple sclerosis

**DOI:** 10.1172/JCI178949

**Published:** 2024-11-01

**Authors:** Federico Fondelli, Jana Willemyns, Roger Domenech-Garcia, Maria José Mansilla, Gerard Godoy-Tena, Anna G. Ferreté-Bonastre, Alex Agúndez-Moreno, Silvia Presas-Rodriguez, Cristina Ramo-Tello, Esteban Ballestar, Eva Martínez-Cáceres

**Affiliations:** 1 Immunology Division, Laboratori Clínic de la Metropolitana Nord, Germans Trias i Pujol University Hospital and Research Institute, Campus Can Ruti, Badalona, Spain.; 2 Department of Cellular Biology, Physiology and Immunology, Universitat Autònoma de Barcelona, Cerdanyola del Vallès, Spain.; 3 Epigenetics and Immune Disease Group, Josep Carreras Leukaemia Research Institute (IJC), Badalona, Barcelona, Spain.; 4 Multiple Sclerosis Unit, Department of Neurosciences, Germans Trias i Pujol University Hospital, Badalona, Spain.; 5 Department of Medicine, Campus Bellaterra, Universitat Autònoma de Barcelona, Cerdanyola del Vallès, Spain.; 6 Epigenetics in Inflammatory and Metabolic Diseases Laboratory, Health Science Center (HSC), East China Normal University (ECNU), Shanghai, China.

**Keywords:** Autoimmunity, Therapeutics, Demyelinating disorders, Dendritic cells, Immunotherapy

## Abstract

Multiple sclerosis (MS) is a chronic disease characterized by dysregulated self-reactive immune responses that damage the neurons’ myelin sheath, leading to progressive disability. The primary therapeutic option, immunosuppressants, inhibits pathogenic anti-myelin responses but depresses the immune system. Antigen-specific monocyte-derived autologous tolerogenic dendritic cells (tolDCs) offer alternative therapeutic approaches to restore tolerance to autoantigens without causing generalized immunosuppression. However, immune dysregulation in MS could impact the properties of the monocytes used as starting material for this cell therapy. Here, we characterized CD14^+^ monocytes, mature dendritic cells, and vitamin D_3_–tolDCs (VitD3-tolDCs) from active, treatment-naive MS patients and healthy donors (HDs). Using multiomics, we identified a switch in these cell types toward proinflammatory features characterized by alterations in the aryl hydrocarbon receptor (AhR) and NF-κB pathways. MS patient–derived VitD3-tolDCs showed reduced tolerogenic properties compared with those from HDs, which were fully restored through direct AhR agonism and by use of in vivo or in vitro dimethyl fumarate (DMF) supplementation. Additionally, in the experimental autoimmune encephalomyelitis mouse model, combined therapy of DMF and VitD3-tolDCs was more efficient than monotherapies in reducing the clinical score of mice. We propose that a combined therapy with DMF and VitD3-tolDCs offers enhanced therapeutic potential in treating MS.

## Abstract 2

Pharmacological Modulation of AhR and NFkB with Dimethyl fumarate in Vitamin-D3-Tolerogenic Dendritic Cells derived from multiple sclerosis patients restores their properties, providing a promising combined therapy.

## Introduction

Immunosuppressive therapies have greatly contributed to improving the outcome and quality of life of patients with autoimmune diseases. Multiple sclerosis (MS), a neuroinflammatory disease of the central nervous system (CNS) in which autoreactive lymphocytes attack the myelin sheath around neurons, exemplifies the application of immunosuppressive therapies in treating autoimmune diseases. Nowadays, MS patients have access to a great arsenal of first- and second-line drugs able to reduce inflammation and clinical manifestations, from monoclonal antibodies to drugs that inhibit lymphocyte migration to the CNS (1). Nevertheless, most of these drugs need lifelong administration and, most importantly, are nonspecific, meaning they act by depressing the immune system in an attempt to block the underlying pathogenic autoreactive response. In some cases, the sustained immunosuppression achieved with certain therapies may be associated with abrogation of beneficial immunoregulatory networks, as well as an increased risk of secondary infection and cancer (2). Generalized immunosuppression represents a challenge in the treatment of MS and other autoimmune diseases. In this context, new strategies are being developed to eliminate autoimmune responses while avoiding generalized immunosuppression, thereby preserving protective immunity. Among these, tolerogenic dendritic cells (tolDCs) are important actors in the arsenal of antigen-specific cellular therapies aimed at restoring tolerance in autoimmunity and transplantation. Indeed, when loaded with an autoantigen, tolDCs can induce tolerance against this self-antigen, re-educating the autoreactive immune system toward homeostasis. This is achieved through different mechanisms, including induction of T cell deletion, anergy, and regulatory T cells (Tregs), which might be able to trigger long-lasting tolerogenic circuits (3). Therefore, loading tolDCs with myelin antigens could enable the specific targeting of pathogenic, myelin-specific autoreactive T cells.

The tolerogenic capacity of tolDCs in autoimmunity and transplantation has been extensively studied in vitro (4–6) and in different animal models (7–12). These studies provided proof of concept for the use of tolDCs on humans and led to clinical trials investigating their application in autoimmune diseases (3). In this context, our group developed an autologous tolDC-based therapy using peripheral blood CD14^+^ monocytes differentiated in the presence of the tolerizing agent vitamin D_3_ (VitD3-tolDCs) and loaded with a myelin peptide pool. This cell therapy has been administered in 2 harmonized, dose-escalation, phase I clinical trials involving active relapsing-remitting MS patients (NCT02903537, NCT02618902, ClinicalTrials.gov), demonstrating safety and feasibility.

While the results from clinical experimentation are promising, data indicating clinical amelioration and tolerance induction are not definitive. This could be due to the small size of the existing trials and the current lack of information about the optimal route of administration and posology. Other critical elements for the design of successful tolDC therapies are the identification of unambiguous biomarkers of tolerance induction, and the generation of functional tolDCs with sufficient potency for in vivo efficacy. The latter is crucial, as these cells must efficiently exert their tolerance-inducing function during a brief lifespan, which might involve the induction of the long arm of tolerance, represented by Tregs.

Innate immune cells, like monocytes, are altered in autoimmune diseases such as systemic lupus erythematosus, type 1 diabetes, and inflammatory bowel disease (13–17). This is also true of MS, in which there are changes in proportions and absolute numbers of monocytes, as well as alterations in their transcriptome, epigenome, metabolome, and functional properties (18–21), which might be due to the inflammatory environment in which these cells persist. In line with this, the inflammatory status of patients is of particular interest in the manufacturing of monocyte-derived tolDC therapies. Indeed, inflammation-primed monocytes obtained from MS patients could potentially be resistant to tolerizing protocols, resulting in less potent tolDC-based therapies. This hypothesis is supported by studies highlighting transcriptional, epigenetic, and functional differences in tolDCs obtained from MS patients versus those differentiated from healthy individuals (22–24). In particular, DNA methylation is an epigenetic mark of key importance in myeloid cell plasticity, differentiation, transcription factor activity, and function (25, 26), and its alterations have been tightly linked to inflammation (27) and autoimmune diseases like MS (28, 29).

In the present study, we used multiomic and immunological profiling to characterize the properties of monocytes, immunogenic DCs, and tolerogenic VitD3-tolDCs from active, treatment-naive MS patients, in comparison with healthy individuals. Our study has revealed a proinflammatory signature in MS-derived monocytes and their products, with marked alterations in the NF-κB and aryl hydrocarbon receptor (AhR) pathways, which upon reversion improve their tolerogenicity and facilitate the design of a more potent second-generation VitD3-tolDC therapy.

## Results

### Monocytes from MS patients are characterized by an activated phenotype.

**Figure 1:**
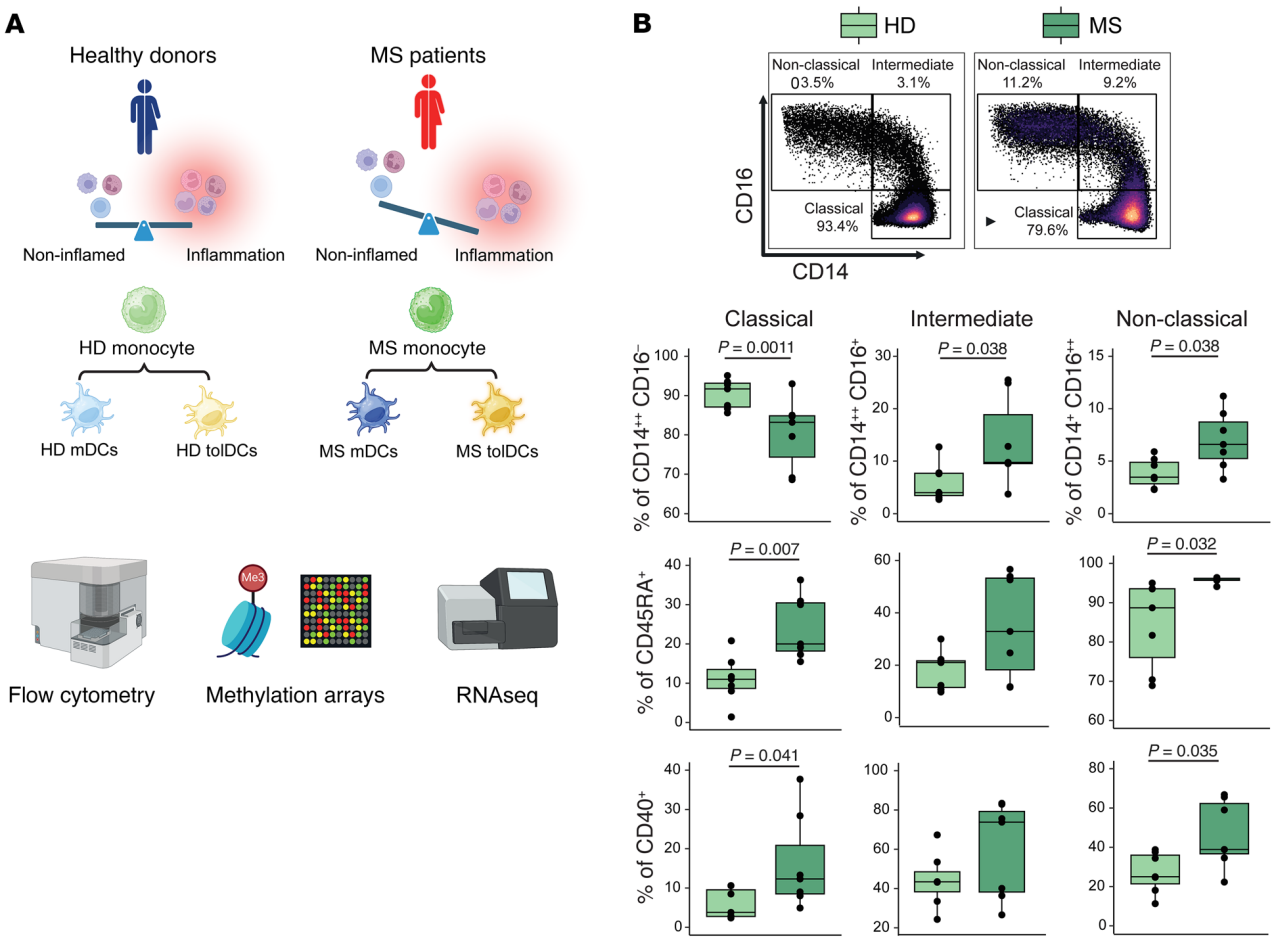
Outline of the study and flow cytometry analysis of MS monocytes. (**A**) Schematic overview of the experimental model from MS- or HD-derived peripheral blood monocytes, mDCs, and tolDCs. Created with BioRender (biorender.com). (**B**) Flow cytometry representative figures and box plots reporting different percentages of classical (CD14^++^CD16^–^), intermediate (CD14^+^CD16^+^), and non-classical (CD14^+^CD16^++^) monocytes among MS patients and HDs, with respect to total monocytes as parent gate (top row), or reporting the percentage of CD45RA^+^ (middle row) or CD40^+^ (bottom row) with respect to classical, intermediate, or non-classical monocytes. *P* values from Mann-Whitney tests are shown in cases of statistical significance. *n* = 7 in each sample group.

To investigate the potential effect of systemic inflammation on circulating monocytes of MS patients, we first studied the peripheral blood CD14^+^ fraction isolated from naive, active relapsing-remitting MS patients and healthy donors (HDs) using spectral flow cytometry and a multiomic approach involving both DNA methylation arrays and bulk RNA sequencing (RNA-Seq) (Figure 1A). Flow cytometry analysis showed an increase in the percentages of non-classical (CD14^+^CD16^++^) and intermediate monocytes (CD14^++^CD16^+^) at the expense of classical monocytes (CD14^++^CD16^–^) in MS patients (Figure 1B). This shift in monocyte subsets was accompanied by higher median fluorescent index of the surface markers CD45RA and CD40 in classical and non-classical subsets (Figure 1B), both of which are activation markers that increase in monocytes in other inflammatory conditions (30–33).

**Figure 2:**
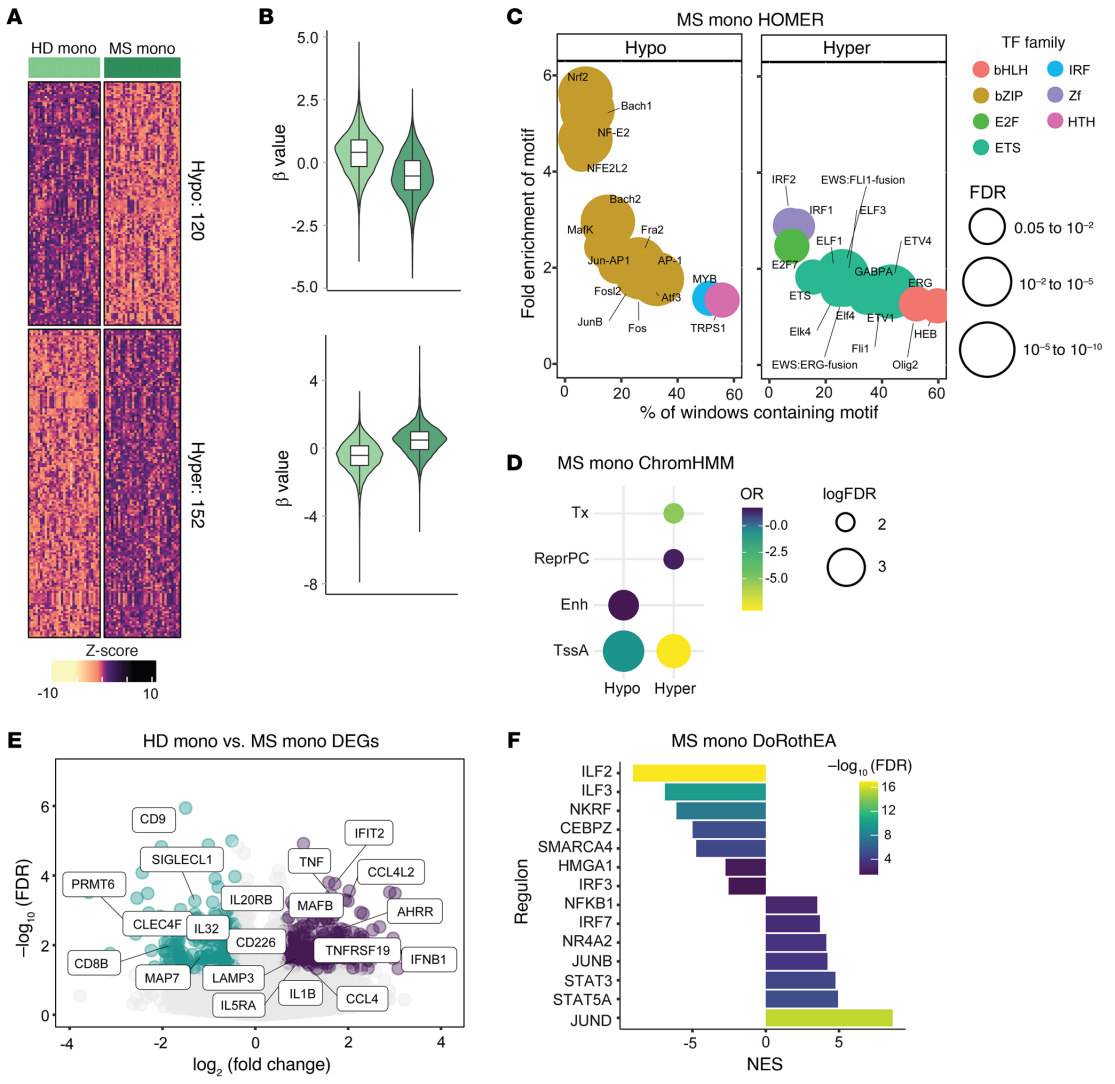
Multiomic characterization of peripheral CD14^+^ cells in MS patients and HDs. (**A**) DNA methylation heatmap of 18 versus 18 samples of HD and MS monocytes (mono). The heatmap includes all CpG-containing probes displaying significant methylation changes (differentially methylated positions [DMPs]) (FDR < 0.05, β > 0.05) in the HD mono–MS mono contrast. (**B**) Violin plots showing the general distribution of DNA methylation across clusters of hyper- or hypomethylation in the HD mono–MS mono contrast. Clear green violin plots correspond to HD mono; dark green violin plots correspond to MS mono. (**C**) Bubble scatterplot showing HOMER analysis of significantly enriched transcription factor (TF) motifs in hypermethylated and hypomethylated cluster regions in HD–MS mono contrast. The *x* axis shows percentage of windows containing the motif, while the *y* axis shows fold enrichment of the motif over background. Colors of bubbles indicate different TF families, while their size is proportional to the FDR. (**D**) Chromatin functional state enrichment analysis of differentially hyper- and hypomethylated probes in the HD mono–MS mono contrast based on ChromHMM public data on CD14^+^ primary cells from the Roadmap Epigenomics project. Odds ratio (OR) is reported on a color scale; sizes of bubbles are proportional to log of FDR. Significantly enriched categories are shown (FDR < 0.05, OR > 2), including strong transcription (Tx), repressed Polycomb (ReprPC), enhancers (Enh), and active transcription starting site (TssA). (**E**) Volcano plots of gene expression showing HD mono–MS mono contrast, with binary logarithm of fold change on the *x* axis and negative decimal logarithm of FDR on the *y* axis. Differentially downregulated and upregulated genes are shown if FDR < 0.05 and fold change < –0.5 or > 0.5. (**F**) Bar plot depicting TF activity predicted from mRNA expression of target genes with DoRothEA v2.0 in the HD–MS mono contrast in terms of normalized enrichment score (NES). Regulons with a high confidence score of A–B were analyzed. A and B refer to benchmarked dataset of curated lists of regulons (list A and list B). A and B are the ones with highest confidence that were used here to estimate transcription factor activity (107). Cases with *P* < 0.05 and NES > 2 and NES < 2 were considered significantly enriched.

Given the critical role of DNA methylation in relation to disease activity in inflammatory diseases (25, 27–29), we profiled DNA methylation of CD14^+^ monocytes obtained from MS patients (MS monocytes) and HDs (HD monocytes).The comparison between MS and HD monocytes showed the existence of differentially methylated positions (DMPs) comprising 120 hypomethylated and 152 hypermethylated positions (false discovery rate [FDR] < 0.05 and absolute differential β (Δβ) > 0.05) (Supplemental Table 1 and Figure 2, A and B), supporting that the DNA methylation profiles of monocytes are also affected in MS. Functional Gene Ontology (GO) analysis (Supplemental Figure 1A; supplemental material available online with this article; https://doi.org/10.1172/JCI178949DS1) of the hypermethylated DMP cluster showed significant enrichment of categories linked to immune response–activating cell surface receptor signaling pathway, antigen presentation, and T and B cell receptor signaling pathway, while the hypomethylated DMP cluster was mainly represented by pathways linked to positive regulation of humoral immunity (Supplemental Figure 1B). Remarkably, flow cytometry analysis did not show changes in HLA-DR median fluorescent index or percentages of HLA-DR^+^ cells among HD and MS monocytes (Supplemental Figure 1C).

Next, we checked for enrichment of transcription factor (TF) binding motifs spanning 250 bp in each direction from differentially hyper- or hypomethylated DMPs (Figure 2C) using HOMER (34). The hypermethylated cluster was enriched in binding motifs of TFs linked to type I interferon response and inflammasome (IRF1, IRF2), immune cell differentiation (ERG), and transcriptional regulation (ETV2, ETV4). ETV2 and ETV4 belong to the same TF family as ETV3 and ETV6, which are crucial in determining IFN responses and fate commitment to monocyte-derived DCs versus monocyte-derived macrophages (35). Moreover, ETV6 is a therapeutic target in the experimental autoimmune encephalomyelitis (EAE) mouse model (35). The hypomethylated DMP cluster was enriched in the binding motifs of TFs regulating NF-κB signature (JunB, Fosl, AP-1), IFN-β production in innate immune cells (ATF3), and NRF2 (NRF2, NFE2LF), a TF induced by metabolic or oxidative stress triggered by inflammation (36), which positively regulates the expression of antiinflammatory molecules.

We then profiled the association of hyper- and hypomethylated DMPs at 18 distinct chromatin states using ChromHMM (37). We observed a significant enrichment of regions of active transcription start sites and enhancers (Figure 2D) in the hypomethylated DMPs, and of active transcription start sites and repressors in the hypermethylated DMPs, suggesting a connection of methylation status and the transcription of genes associated with hypomethylated CpGs. Overall, MS monocytes presented an altered DNA methylation profile, skewed toward a proinflammatory and activated profile.

Bulk RNA-Seq data (Supplemental Table 2 and Figure 2E, left, downregulated genes; right, upregulated genes) supported the acquisition of a transcriptomic signature in MS monocytes compatible with a proinflammatory phenotype. The comparison of the RNA-Seq profiles between MS and HD showed 333 overexpressed and 248 downregulated genes (log fold change < –0.5 or > 0.5, FDR < 0.05). These include the upregulation of inflammation-related genes such as *TNF*, *IFNB1*, *CCL4*, and *AHRR*, encoding the repressor of the aryl hydrocarbon receptor (AhR), a key TF in the acquisition of the tolDC phenotype. Moreover, we observed downregulation of the methyltransferase *PRMT* and *MAP7*, a molecule previously described by our group as a biomarker of VitD3-tolDCs. Finally, we identified TFs potentially involved in the transcriptomic changes observed in MS monocytes by using Discriminant Regulon Expression Analysis (DoRothEA) (38) in our data set. MS monocytes were enriched in several pivotal inflammatory factors (Figure 2F), such as NF-κB, STAT5A, and IRF7. Interestingly, MS monocytes also showed a significant depletion of NFKB repressing factor (NKRF), and of ILF2 and ILF3, which are involved in suppressing the acquisition of a mature phenotype in the monocyte-to-DC axis (39). In conclusion, multilayer analysis of protein expression, transcriptome, and epigenome determined that MS monocytes display a proinflammatory phenotype in comparison with HD monocytes, defined by increased activation of inflammation pathways.

### The proinflammatory signature is maintained in monocyte-derived mature DCs from MS patients.

**Figure 3:**
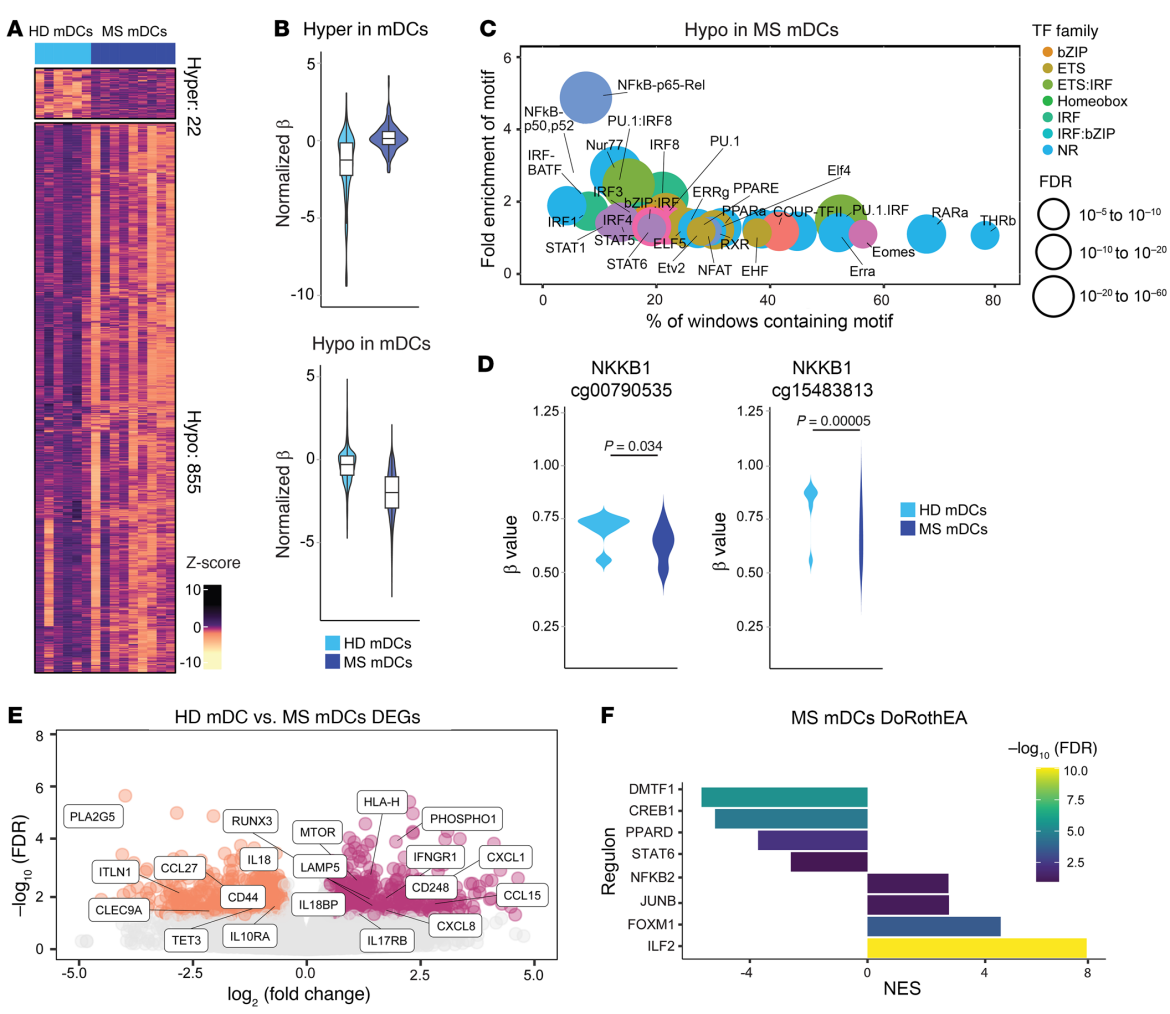
The proinflammatory signature is maintained in monocyte-derived mDCs and tolDCs from MS patients. (**A**) DNA methylation heatmap of 6 versus 8 samples of HD and MS mDCs. The heatmap includes all CpG-containing probes displaying DMPs (*q* value < 0.05, β > 0.05) in the HD mDCs–MS mDCs contrast. (**B**) Violin plots showing the general distribution of DNA methylation across hyper- or hypomethylated clusters in HD mDCs and MS mDCs. (**C**) Bubble scatterplot showing HOMER analysis of significantly enriched TF motifs in the hypermethylated and hypomethylated cluster regions in HD–MS mDCs contrast. (**D**) Violin plots showing DNA methylation levels (β values) of *NFKB1* individual CpGs in HD mDCs–MS mDCs comparisons. *P* values correspond to FDR (significant if FDR < 0.05). (**E**) Volcano plots of gene expression showing HD–MS mDCs contrast, with binary logarithm of fold change on the *x* axis and negative decimal logarithm of FDR on the *y* axis. Differential expression of genes was calculated as described earlier. (**F**) Bar plot depicting the TF activity predicted from mRNA expression of target genes with DoRothEA v2.0 in the HD–MS mDCs contrast in terms of NES. Enriched regulons were identified as described earlier.

To test our hypothesis that MS-intrinsic imprinting on CD14^+^ monocytes is retained after differentiation into monocyte-derived DCs, we also conducted DNA methylation profiling and bulk RNA-Seq of HD- and MS-derived mature DCs (mDCs) and tolDCs. mDCs and tolDCs from MS patients and HD monocytes were differentiated in vitro in either the absence or the presence of vitamin D_3_ as a tolerizing agent. The DNA methylation profiles of MS mDCs displayed differences in comparison with HD mDCs (Supplemental Table 3 and Figure 3, A and B) that mainly consisted of a large cluster of hypomethylation (hypomethylated DMPs = 916; hypermethylated DMPs = 57; FDR < 0.05 and Δβ > 0.05).

Like with MS monocytes, HOMER analysis of MS mDC hypomethylated DMPs showed enrichment of binding motifs of key inflammatory TFs such as NF-κB, p65, STAT1, STAT5, STAT6, IRF1, IRF3, and IRF4, suggesting a more activated phenotype of MS-derived mDCs (Figure 3C). Specifically, we detected hypomethylation of 2 CpGs mapping at the *NFKB1* gene (Figure 3D). On the other hand, there was no significant enrichment of TF binding motifs in the hypermethylated DMP cluster. Functional GO analysis (Supplemental Figure 2A) of the hypomethylated cluster showed enrichment of categories linked to activation of the adaptive immune response. In addition, ChromHMM pointed out enrichment in active transcription start sites, enhancers, and repressors for the hypomethylated DMPs (Supplemental Figure 2B). RNA-Seq data (Supplemental Table 4 and Figure 3E, left, downregulated genes; right, upregulated genes in HD mDCs vs. MS mDCs) also revealed an increase in inflammatory pathways: *CXCL1*, *IL-8*, and *IL-27* genes were upregulated, encoding 3 cytokines that dictate inflammatory responses and are regulated by NF-κB signaling (40–44), as was *mTOR*, which plays a central role in regulating DC differentiation, immune responses, and autophagy (45). On the other hand, MS mDCs expressed less *CD300LB*, a molecule regulating DC efferocytosis (46), *IL-18*, a cytokine inducing Th1 responses (47), and *CLEC9A*, a C-type lectin receptor involved in antigen uptake (48). Finally, MS mDCs were positively enriched in NF-κB and ILF2, a factor linked to the regulation of IL-2 production, and negatively enriched in PPARD, encoding the receptor of PPARγ, which is involved in inducing Th2 responses (Figure 3F). NF-κB signature was increased in MS mDCs according to HOMER and DoRothEA, but this was not reflected in higher *NFKB1* and *TNF* transcript levels in the RNA-Seq data set (Supplemental Figure 2C). Overall, MS mDCs have a more immunogenic profile in comparison with HD, mainly characterized by the activation of the NF-κB pathway.

### Vitamin D tolerization does not reverse MS DCs’ inflammatory fingerprint.

**Figure 4:**
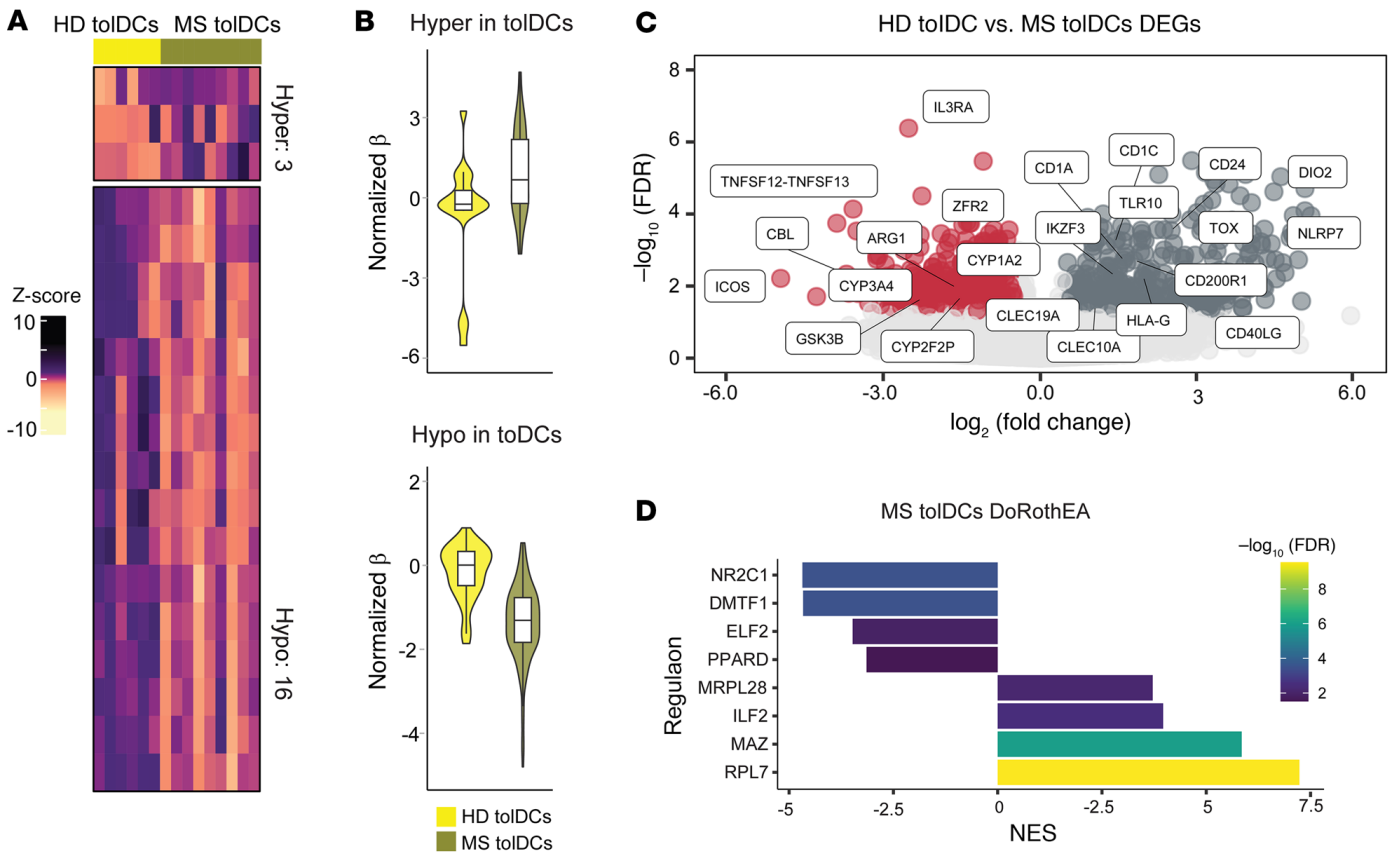
Vitamin D tolerization does not reverse MS DCs’ inflammatory fingerprint. (**A**) DNA methylation heatmap of 6 versus 8 samples of HD and MS tolDCs. The heatmap includes all CpG-containing probes displaying DMPs (FDR < 0.05, β > 0.05) in the HD tolDCs–MS tolDCs contrast. (**B**) Violin plots showing the general distribution of DNA methylation across hyper- or hypomethylated clusters in HD tolDCs and MS tolDCs. (**C**) Volcano plots of gene expression showing HD–MS tolDCs contrast, with binary logarithm of fold change on the *x* axis and negative decimal logarithm of FDR on the *y* axis. Differential expression of genes was calculated as described earlier. (**D**) Bar plot depicting the TF activity predicted from mRNA expression of target genes with DoRothEA v2.0 in the HD–MS tolDCs contrast in terms of NES. Enriched regulons were identified as described earlier.

In contrast with MS mDCs, MS tolDCs did not show wide DNA methylation changes in comparison with HD tolDCs (Supplemental Table 3 and Figure 4, A and B), with very few DMPs present in this comparison. On the other hand, MS tolDCs still showed changes at the transcriptomic level (Supplemental Table 4 and Figure 4C), with an increased expression of the maturation/activation markers *CD1c*, *CD1a*, and *CD24* and reduced expression of the *CYP1A2* gene. *CYP1A2* is used, together with *CYP1A1*, as a surrogate marker to infer *AHR* activity, which is also involved in monocyte-to-DC differentiation, in addition to the acquisition of tolerogenic features (49–51). Additionally, MS tolDCs expressed less *ARG1*, involved in conferring immunosuppressive properties to tolDCs (52). Regulon analysis using DoRothEA showed a negative enrichment of PPARD and positive enrichment of ILF2, as observed in MS mDCs (Figure 4D). Taken together, these results indicate that despite the few differences at the DNA methylation level, MS tolDCs appear to have a more mature and activated transcriptomic profile in comparison with HD tolDCs.

### MS monocytes, mDCs, and tolDCs share alterations in the AHR pathway.

**Figure 5:**
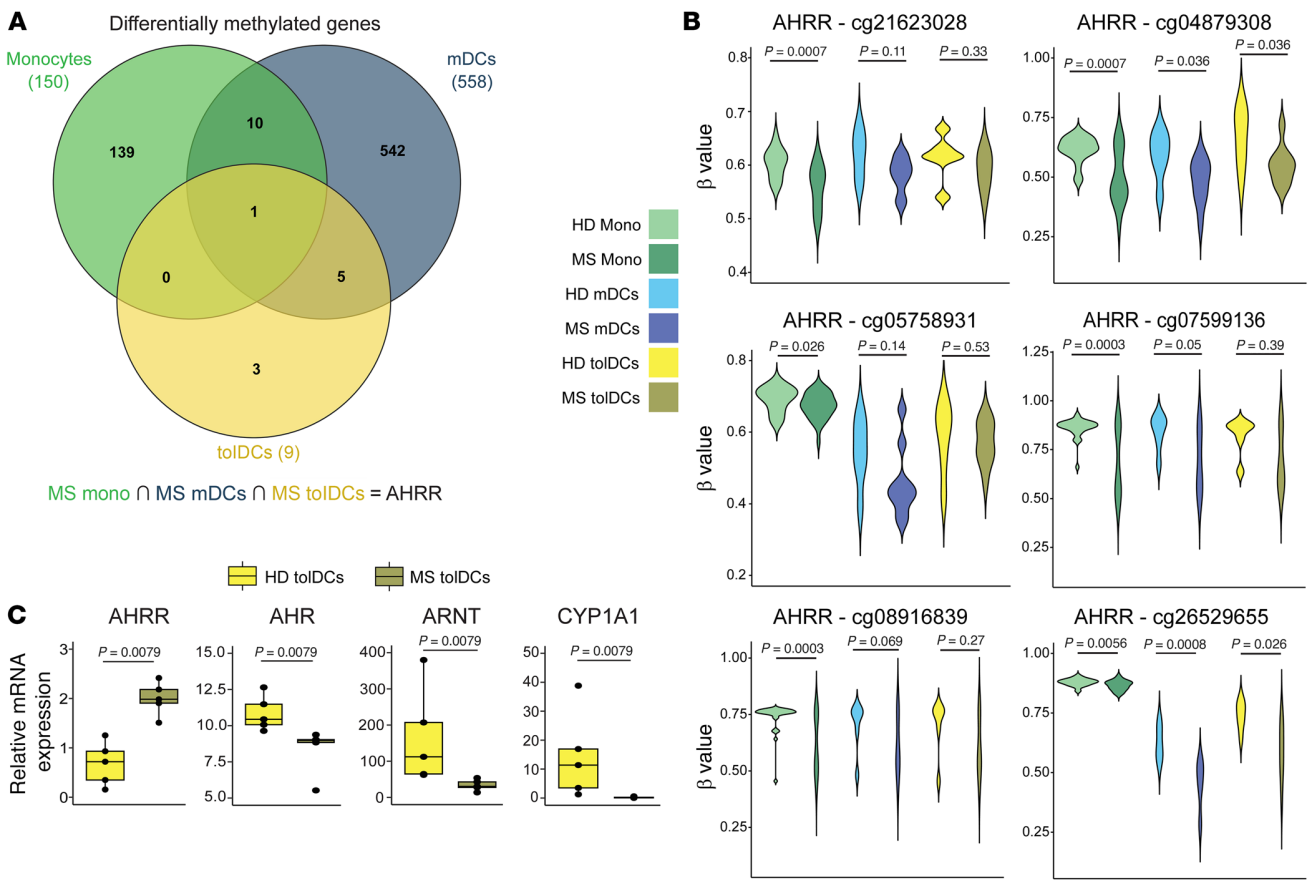
MS Mono, mDCs, and tolDCs share alterations in the AHR pathway. (**A**) Venn diagram showing shared hyper- and hypomethylated genes linked to significant differential methylation changes (DMPs) across HD-MS contrasts, in different cell types (MS mono, MS mDCs, and MS tolDCs). (**B**) Violin plots showing DNA methylation levels (β values) of *AHRR* individual CpGs in hypermethylated and hypomethylated sets across all 3 comparisons. *P* values correspond to FDR (significant if FDR < 0.05). (**C**) Box plots of relative expression of individual genes performed by reverse transcriptase qPCR (RT-qPCR) of mRNA in HD tolDCs–MS tolDCs. *P* values from Mann-Whitney tests are shown. *n* = 5 per sample group, 2 independent experiments.

To identify pathways that are altered in MS monocytes and whose dysregulation persists across the in vitro differentiation to MS mDCs or MS tolDCs, we inspected common DMPs and DEGs across the 3 different cell types. In relation to DNA methylation (Figure 5A), after annotating DMPs to the single nearest gene, we found that only 1 differentially methylated gene was shared across the 3 cell types, annotating to *AHRR*. Specifically, *AHRR* was hypomethylated in MS cell types versus HD at the level of 6 different CpGs, with statistical significance depending on the specific CpG and cell type (Figure 5B).

In relation to the occurrence of common transcriptomic changes, MS monocytes, mDCs, and tolDCs shared upregulation of *PPBP*, which is associated with positive regulation of immunity (53), and of *MSLN* and *PKHD1L1*, whose role in innate immunity is not known (Supplemental Figure 3A). No shared differentially downregulated genes were found across the 3 cell types (Supplemental Figure 3B).

In addition to the changes in the *AHRR* methylation levels, MS monocytes expressed more *AHRR*, while MS tolDCs expressed less *CYP1A2*, suggesting the possible occurrence of differences in the AHR pathway in MS monocytes and derived cells. To validate this hypothesis in MS tolDCs, we quantitated the transcript levels of *AHRR*, *ARNT*, *AHR*, and *CYP1A1* in tolDCs from 2 additional cohorts of MS patients and HDs. *ARNT* encodes the AhR translocator protein, and *CYP1A1* is an AHR target that can be used as a surrogate of AHR activity. MS tolDCs showed higher mRNA levels of *AHRR* and lower levels of *ARNT* and *AHR* (Figure 5C). In line with this, *CYP1A1* expression was higher in HD tolDCs than in MS tolDCs (Figure 5C). Overall, the AHR pathway was dysregulated in MS tolDCs at the level of gene expression and DNA methylation.

### Modulation of the AHR pathway influences the tolerogenic profile of tolDCs.

**Figure 6:**
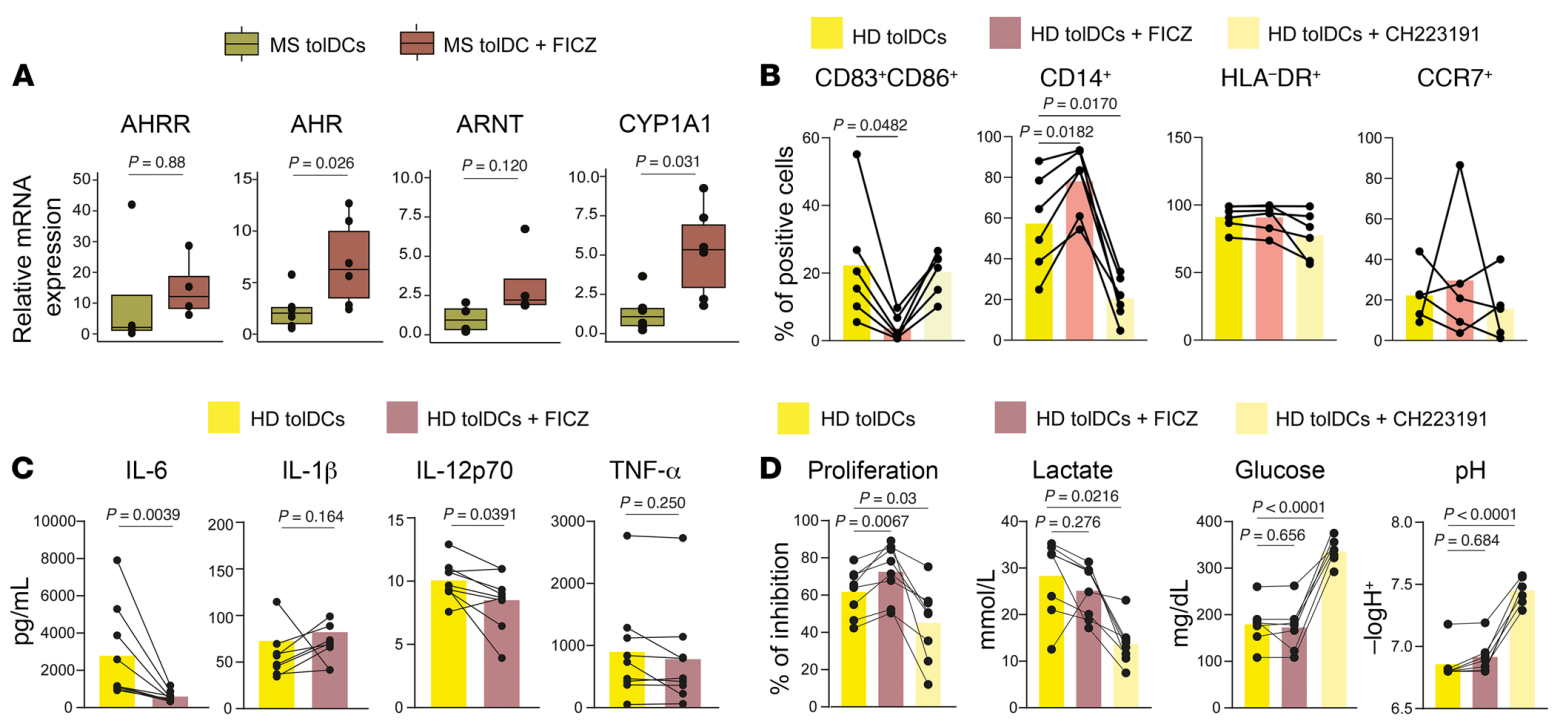
Modulation of the AHR pathway influences the tolerogenic profile of tolDCs. (**A**) Box plots of relative expression of individual genes performed by RT-qPCR of mRNA in HD tolDCs versus tolDCs + FICZ. *P* values from Wilcoxon’s tests are shown. *n* = 4–6 depending on the gene, 2 independent experiments. (**B**) Before-after scatter bar plot showing flow cytometry data relative to the percentage of CD83^+^CD86^+^, CD14^+^, CCR7^+^, or HLA-DR^+^ cells among tolDCs, tolDCs + FICZ, and tolDCs + CH223191. *P* values from repeated-measures 1-way ANOVA with multiple comparisons are shown. *n* = 6 in each sample group. (**C**) Before-after scatter bar plot representing the effect of FICZ agonist on production of IL-6, IL-12p70, and IL-1β cytokines by tolDCs. FICZ was added at day 0 and day 4 of differentiation of tolDCs, with a final concentration of 18 μM. *P* values from Wilcoxon’s tests are shown. *n* = 9 in each sample group. (**D**) Proliferation of allogeneic peripheral mononuclear cells cocultured with HD tolDCs and tolDCs differentiated in the presence of either FICZ (HD tolDCs + FICZ) or CH223191 (HD tolDCs + CH223191). Inhibition of proliferation was assessed as percentage of Violet 450–positive lymphocytes and calculated using mDC-induced proliferation as reference for each sample by the following formula: (mDCs – tolDCs)/mDCs, obtaining the percentage of reduction of proliferation of tolDCs in reference to the donor-matched mDCs. *P* values from repeated-measures 1-way ANOVA with multiple comparisons are shown. *n* = 8 in each sample group. (**E**) Quantification of pH, glucose, and lactate concentration on day 6 cell culture supernatants. *P* values from repeated-measures 1-way ANOVA with multiple comparisons are shown. *n* = 8 in each sample group.

To prove that AHR is implicated in the acquisition of the tolerogenic program of our cell therapy, we differentiated VitD3-tolDCs in the presence of a specific agonist (FICZ) or an inhibitor (CH223191) of AHR and evaluated their effects on gene expression and functionality. First, the AHR agonist FICZ induced increased expression of the *AHR* gene and *CYP1A1* in MS tolDCs, supporting the occurrence of activation of the pathway (Figure 6A). On the other hand, FICZ agonism did not induce any significant change in the expression of *AHRR* and *ARNT*. AHR agonism increased the percentages of CD14^+^ tolDCs and reduced the CD83^+^CD86^+^ population, while antagonism reduced CD14^+^ cells (Figure 6B). No significant changes in HLA-DR and CCR7 were observed using the agonist or antagonist. In addition, HD tolDCs differentiated with FICZ produced less IL-6 and IL-12p70 (Figure 6C). This effect is supported by functional data obtained by allogeneic mixed lymphocyte reaction (MLR), in which HD tolDCs differentiated in the presence of FICZ were less able to induce allogeneic PBMC proliferation in comparison with conventional tolDCs, while tolDCs differentiated in the presence of CH223191 induced more proliferation (Figure 6D). Finally, AHR antagonism induced an increase in the pH of the medium and a reduction in both glucose consumption and lactate production (Figure 6E). Glycolysis is a hallmark of VitD3-tolDC metabolism (54), and lactate plays an important role in defining their tolerogenic function (4, 55). Taken together, these results led us to hypothesize that AHR is partially implicated in defining VitD3-tolDC functionality and that agonism of this pathway induced a more immature and tolerogenic phenotype.

### In vitro dimethyl fumarate supplementation boosts VitD3-tolDC tolerogenicity.

While AHR agonism with FICZ improved MS tolDC tolerogenic features, its clinical use in MS is challenging owing to its instability, rapid pharmacokinetics (56), and induction of Th17 cells, which drive MS pathogenesis (57). In contrast, dimethyl fumarate (DMF), an approved oral treatment for relapsing-remitting and active secondary progressive MS, has immunomodulatory effects and a good tolerability profile. DMF strongly activates NRF2 and inhibits NF-κB (58, 59), mimicking AHR agonism in myeloid cells, and can upregulate AHR pathways directly and indirectly through NRF2 (60, 61). Therefore, we investigated the effects of DMF on tolDC gene expression, metabolism, and functionality as a potential AHR agonist surrogate and NF-κB inhibitor.

**Figure 7:**
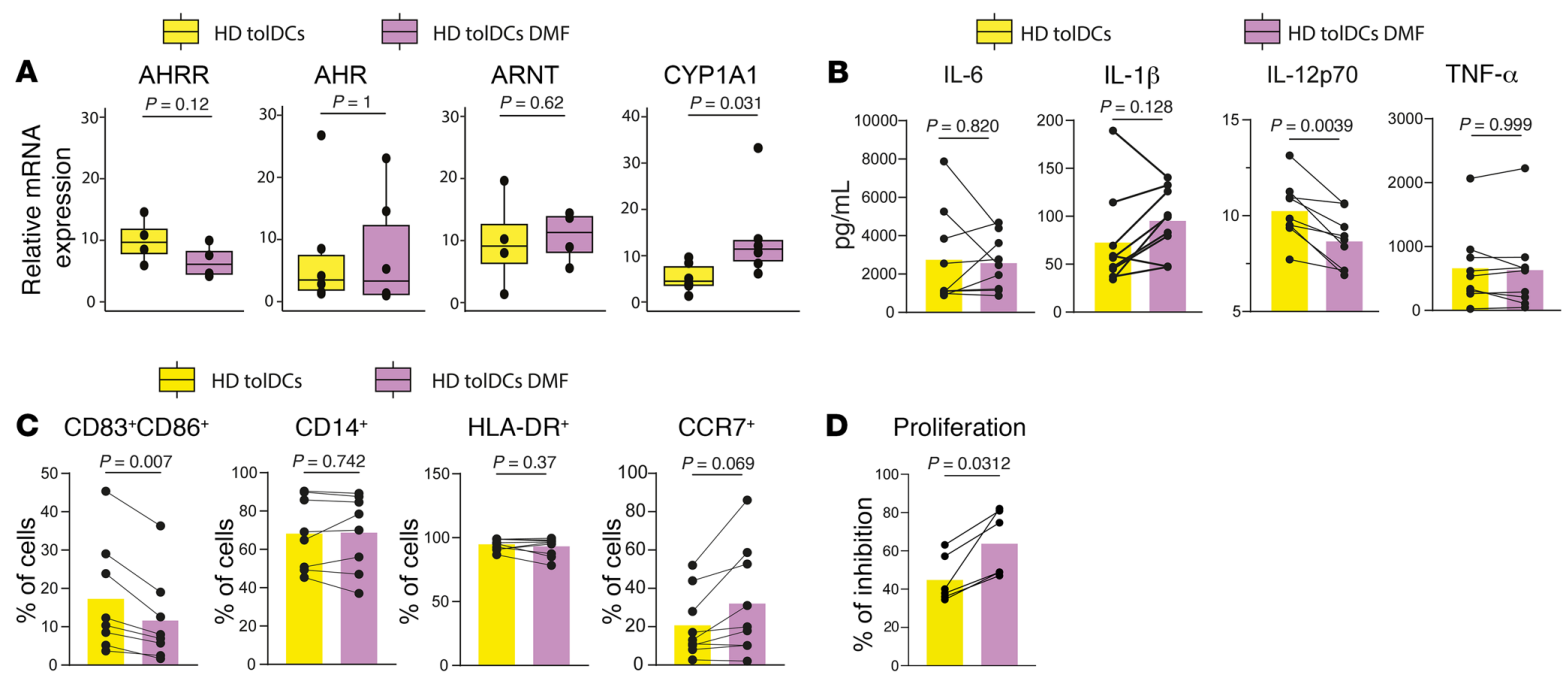
In vitro DMF supplementation increases VitD3-tolDC tolerogenicity. (**A**) Box plots of relative expression of individual genes performed by RT-qPCR of mRNA in HD tolDCs versus HD tolDCs + DMF. DMF was added at day 0 and day 4 of differentiation of tolDCs, with a final concentration of 10 μM. *P* values from Wilcoxon’s tests are shown. *n* = 4–6 depending on the gene analyzed. (**B**) Before-after scatter bar plot representing the effect of DMF on production of IL-6, IL-12p70, and IL-1β cytokines by tolDCs. TolDC HD data were already presented in Figure 3F. *P* values from Wilcoxon’s tests are shown. *n* = 9 in each sample group. (**C**) Before-after scatter bar plot showing flow cytometry data relative to the percentage of CD83^+^CD86^+^, CD14^+^, HLA-DR^+^, and CCR7^+^ cells among HD tolDCs and HD tolDCs DMF. *P* values from Wilcoxon’s tests are shown. *n* = 8 per sample group. (**D**) Proliferation of allogeneic peripheral mononuclear cells cocultured with HD tolDCs and HD tolDCs DMF. Inhibition of proliferation was assessed as described earlier. *P* values from Wilcoxon’s tests are shown. *n* = 6 per sample group.

First, we checked the effect of DMF on the differentiation from HD monocytes to HD tolDCs. Analysis of quantitative PCR (qPCR) data showed that DMF triggered *CYP1A1* expression, while *AHR*, *AHRR*, and *ARNT* transcript levels did not change (Figure 7A). From a functional point of view, HD tolDCs treated with DMF in vitro (HD tolDCs DMF) produced less IL-12p70 in comparison with HD tolDCs (Figure 7B), suggesting a less immunogenic phenotype. Flow cytometry data show lower CD83^+^CD86^+^ percentages in HD tolDCs DMF (Figure 7C). No effects were observed on CD14 and HLA-DR percentages (Figure 7C). Importantly, HD tolDCs differentiated with DMF inhibited more allogeneic proliferation in MLRs in comparison with HD (Figure 7D).

**Figure 8:**
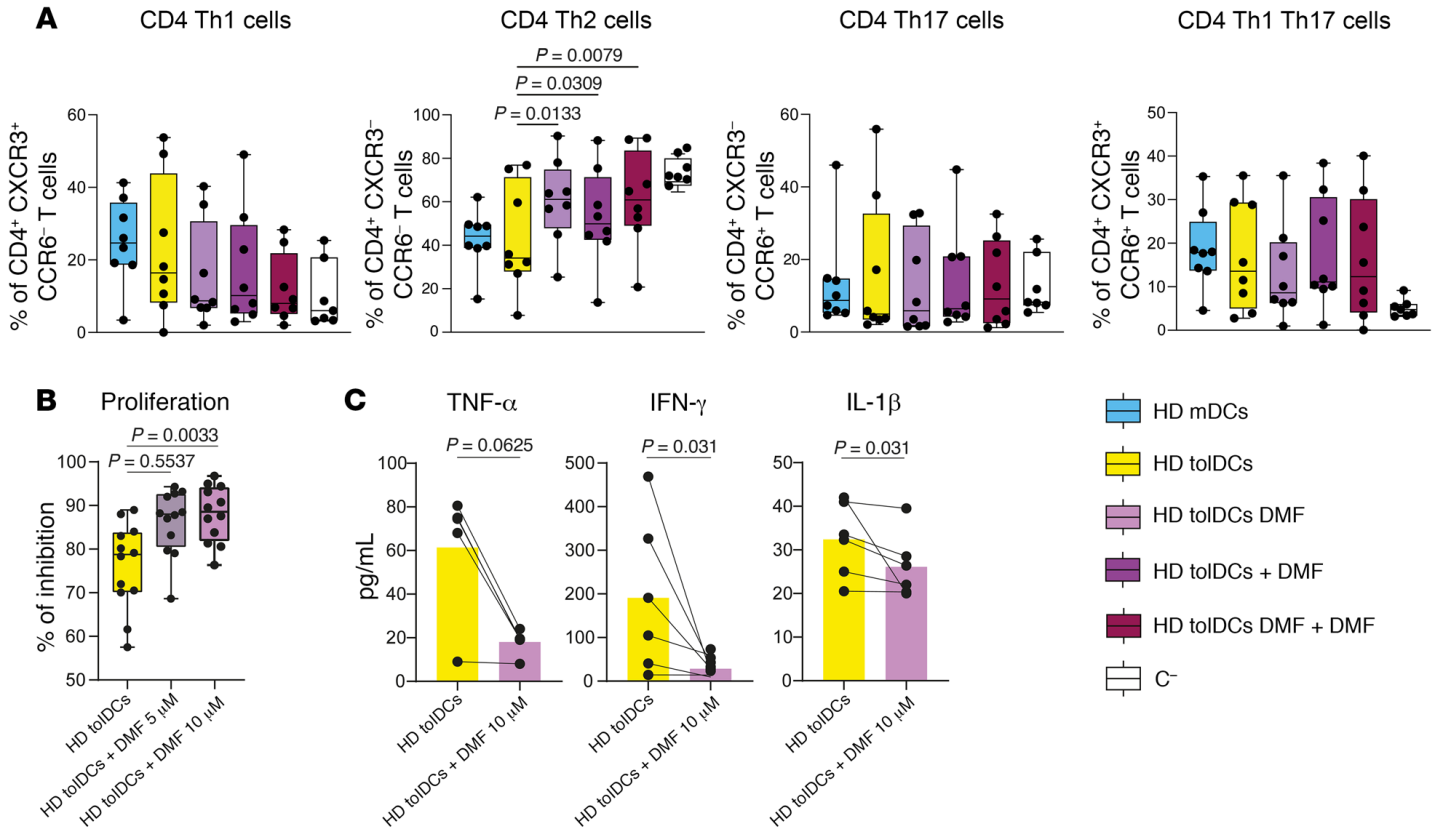
TolDCs + DMF modulate allogeneic PBMC properties in vitro. (**A**) Box plots of percentage of CD4^+^ Th1, Th2, Th17, and Th1Th17 cells analyzed through flow cytometry after 6 days of DC-PBMC allogeneic cocultures. *P* values from ANOVA with multiple comparisons are shown (mixed-effects analysis). *n* = 8 per sample group. Different coculture conditions include PBMCs with HD mDCs, tolDCs, HD tolDCs differentiated in the presence of DMF (HD tolDCs DMF), HD tolDCs with DMF added directly in the coculture (HD tolDCs + DMF), HD tolDCs differentiated in the presence of DMF and for which DMF is added directly in the coculture (HD tolDCs DMF + DMF), and no tolDCs (C^–^). (**B**) Proliferation of PBMCs cocultured with HD tolDCs without or with 5 μM or 10 μM DMF. Inhibition of proliferation was assessed as described earlier. One-way repeated-measures ANOVA with multiple comparisons was used to calculate significant differences among groups, reported as *P* values. *n* = 12. (**C**) Before-after scatter bar plot representing the effect of DMF on production of IFN-γ, TNF-α, and IL-1β by tolDCs. DMF was added during the coculture with HD tolDCs and allogeneic PBMCs at day 0. *n* = 5 or *n* = 6 depending on the sample group. *P* values from Wilcoxon’s tests are shown.

Finally, we studied T cell polarization after HD-derived DC-PBMC cocultures in different experimental conditions with or without DMF. After 6 days of coculture, no differences were observed in the percentages of naive, central memory, effector memory, or terminally differentiated effector memory CD4^+^ T cells among the different groups (Supplemental Figure 4A). On the other hand, there was an increase in the percentage of CD4^+^ T helper type 2 (Th2) in cocultures with HD tolDCs DMF + DMF in comparison with the other groups (Figure 8A) and lower activated CD38^+^ CD4^+^ T cells (Supplemental Figure 4B). Instead, HLA-DR expression was not affected (Supplemental Figure 4C). Finally, addition of 10 μM DMF to tolDC-PBMC allogeneic MLRs determined less proliferation and reduction in IFN-γ and IL-1β production in comparison with HD tolDC alone (Figure 8, B and C). DMF also reduced allogeneic proliferation in mDC-PBMC MLRs (Supplemental Figure 4D). Overall, in vitro supplementation of DMF during the differentiation to tolDCs seems to potentiate their tolerogenic potency. Moreover, DMF also seems to exert effects that are independent of tolDC activity in allogeneic MLRs.

### Administration of DMF to MS patients restores fully functional tolDCs.

**Figure 9:**
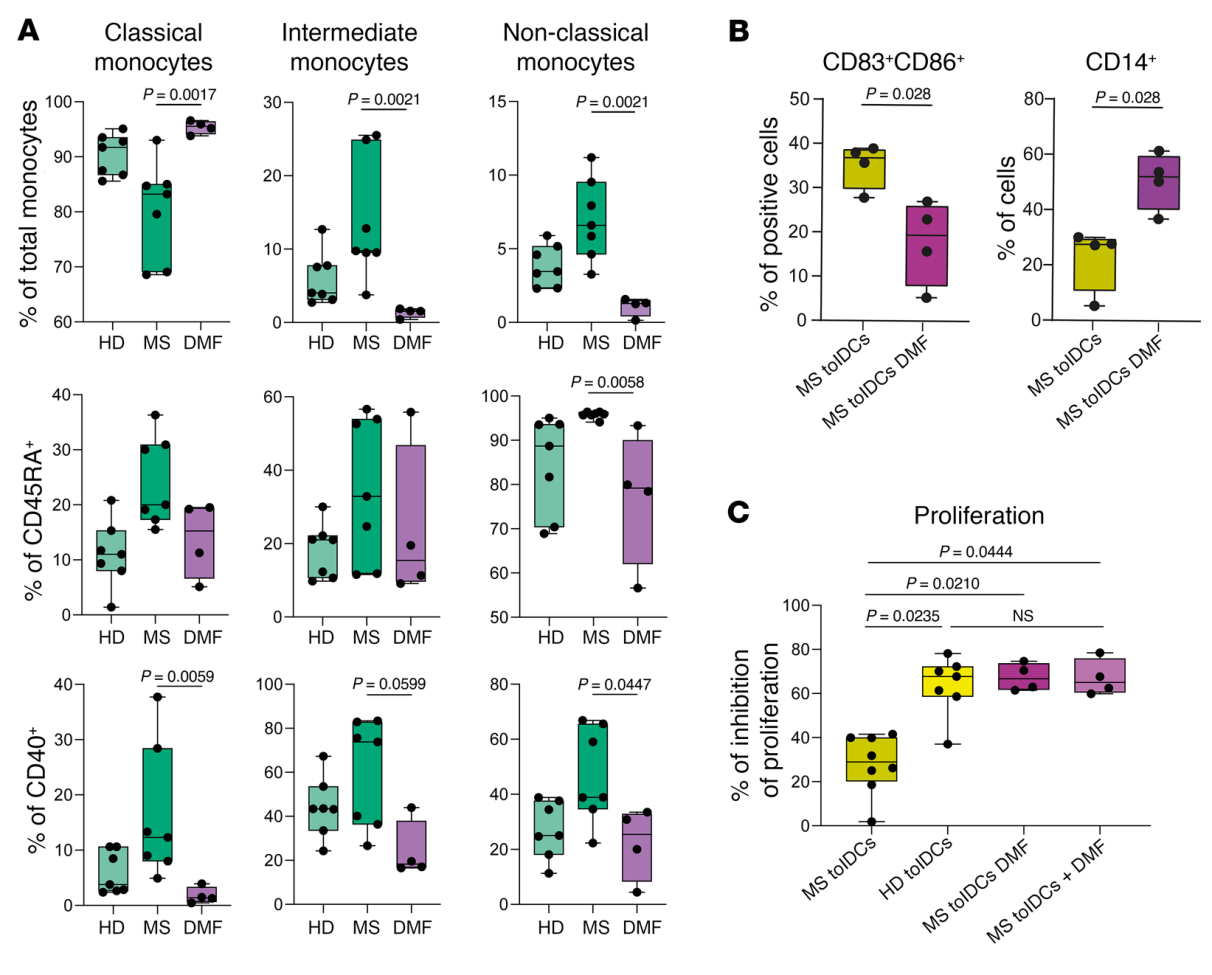
In vivo administration of DMF to MS patients restores fully functional tolDCs. (**A**) Box plots reporting percentages of classical, intermediate, and non-classical monocytes among HD and MS patients without treatment (MS) or treated with DMF (DMF), with respect to total monocytes as parent gate (top row), or reporting percentages of CD45RA^+^ (middle row) or CD40^+^ (bottom row) classical, intermediate, and non-classical monocytes. *P* values from Kruskal-Wallis test with Dunn’s multiple comparisons are shown. *n* = 7 or *n* = 4 depending on the sample group. Percentages of monocyte subpopulations from HD and MS patient groups are also presented in Figure 1B. Here, new statistical tests have been applied to include a cohort of DMF-treated patients. (**B**) Box plots showing flow cytometry data relative to the percentage of CD83^+^CD86^+^ and CD14^+^ cells after 6-day in vitro differentiation among MS tolDCs and among tolDCs isolated from patients undergoing DMF treatment (MS tolDCs DMF). Unpaired, 2-tailed *t* tests were used to calculate *P* values (Mann-Whitney tests). *n* = 4 in each sample group. (**C**) Proliferation of allogeneic PBMCs with tolDCs from HDs, treatment-naive MS patients (MS tolDCs), or patients undergoing DMF treatment (MS tolDCs DMF) or with tolDCs from MS patients differentiated in the presence of DMF in vitro (MS tolDCs + DMF). Inhibition of proliferation was assessed as described earlier. One-way ANOVA with multiple comparisons was used to calculate significant differences among groups (Kruskal-Wallis test), reported as *P* values. *n* = 4–8 depending on the sample group.

Then, we evaluated whether in vivo administration of DMF to MS patients could influence the functionality of MS tolDCs. Firstly, we profiled through spectral flow cytometry the expression of markers in monocytes from a new cohort of MS patients receiving DMF treatment (DMF) and compared the data from the previous cohorts of HD and naive MS patients (MS). Similarly to HD, DMF patients showed higher percentages of classical monocytes and fewer intermediate and non-classical monocytes in comparison with MS patients (Figure 9A, top row). Moreover, DMF reduced the percentages of CD45RA^+^ non-classical monocytes and of CD40^+^ classical and non-classical monocytes (Figure 9A, middle and bottom rows). In comparison with MS patients, classical, intermediate, and non-classical monocytes from DMF patients showed lower median fluorescent index of CX3CR1 (Supplemental Figure 5, top row), a chemokine receptor involved in trafficking to inflammation sites and the CNS in MS (62). A higher median fluorescent index of PD-L1 in intermediate and non-classical monocytes in comparison with MS patients and HDs was also observed (Supplemental Figure 5, bottom row). Secondly, we differentiated tolDCs from monocytes obtained from naive patients (MS tolDCs) and patients receiving DMF treatment (MS tolDCs DMF) and compared their phenotype at day 6 of culture via flow cytometry. MS tolDCs DMF were characterized by a higher expression of CD14 and a decreased CD83^+^CD86^+^ population (Figure 9B). Then, to define the effect of in vivo DMF administration on the functionality of tolDCs, we studied, through allogeneic MLR, tolDCs differentiated from HDs, MS patients (MS tolDCs), MS patients receiving DMF treatment (MS tolDCs DMF), and MS patients with DMF added in vitro during the differentiation (MS tolDCs + DMF). MS tolDCs suppressed less allogeneic PBMC proliferation in comparison with HD tolDCs, and in comparison with MS tolDCs DMF and MS tolDCs + DMF (Figure 9C). On the other hand, MS tolDCs DMF and MS tolDCs + DMF showed an inhibition of allogeneic proliferation that was comparable to that of HD (Figure 9C). Overall, administration of DMF to MS patients seems to induce monocytes with a regulatory profile and allows for the differentiation of tolDCs with HD-like functional profile.

### Combined therapy with DMF and tolDCs has higher clinical potential in comparison with monotherapies.

**Figure 10:**
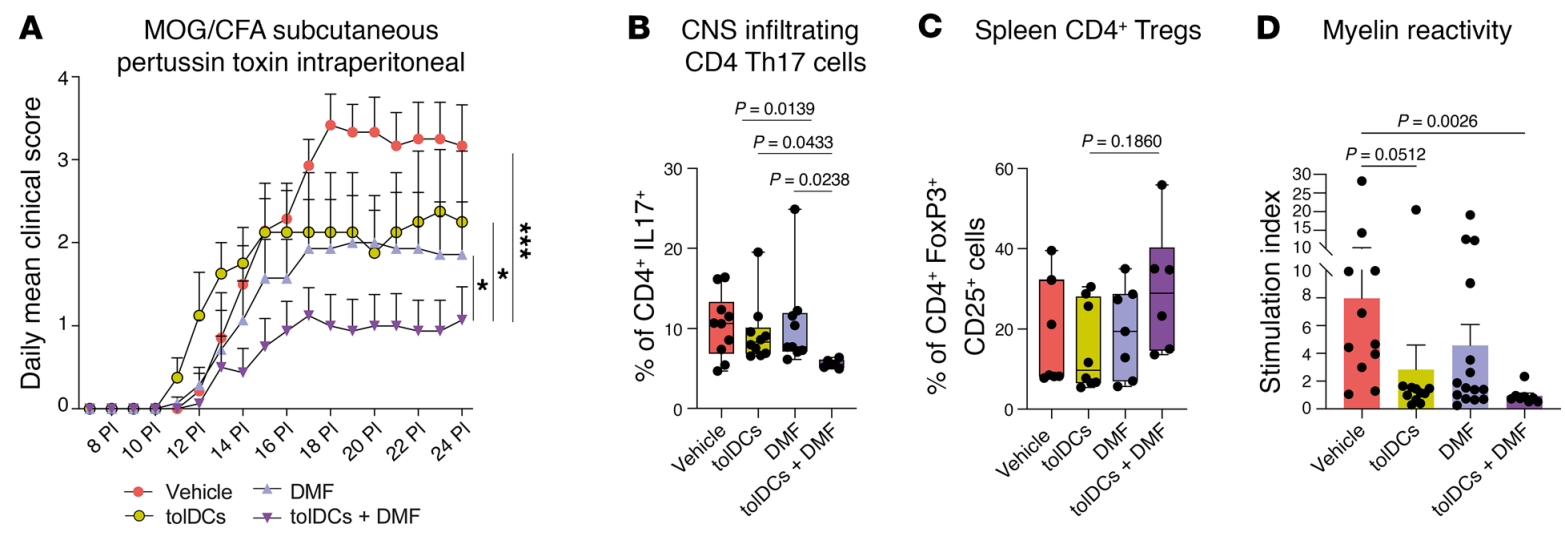
DMF + tolDCs combined therapy has higher clinical potential in comparison with monotherapies. (**A**) Daily mean clinical score of C57BL/6 mice immunized with MOG_35–55_ peptide treated with vehicle (PBS) (red circles, *n* = 7), DMF (lavender triangles, *n* = 7), tolDCs (yellow circles, *n* = 4), or tolDCs + DMF (purple triangles, *n* = 8). *P* values were obtained by Holm-Šidák multiple-comparison test (*P* > 0.05, NS; *P < 0.05, ***P < 0.001). Data are shown as mean ± SEM. Data are from a single mouse experiment. (**B**) Box plots showing the percentage of total CD4^+^ IL-17^+^ cells in the cell infiltrate of spinal cords from mice treated with vehicle (PBS and methylcellulose, *n* = 10), DMF (*n* = 9), tolDCs (*n* = 10), or tolDCs + DMF (*n* = 6) on day 24 post-immunization day (PI). Samples were analyzed through flow cytometry after intracellular and surface staining. *P* values were obtained by Kruskal-Wallis test. Data are from 2 independent experiments. (**C**) Box plots showing the percentage of CD4^+^CD25^+^FoxP3^+^ cells among total CD4^+^ T cells from spleens of mice treated with vehicle (*n* = 7), DMF (*n* = 7), tolDCs (*n* = 8), or tolDCs + DMF (*n* = 6) on day 24 PI. Samples were analyzed through flow cytometry after intracellular and surface staining. *P* values were obtained by Kruskal-Wallis test. (**D**) Analysis of antigen-specific T cell reactivity to MOG_35–55_ in splenocytes from mice treated with vehicle (*n* = 11), tolDCs (*n* = 15), DMF (*n* = 11), or tolDCs + DMF (*n* = 8) on day 24 PI. The mean stimulation index was calculated for each group after 4 days of incubation. Error bars correspond to SEM. *P* values were obtained by Kruskal-Wallis test.

Finally, we assessed the effects of a combined therapy of DMF plus tolDCs in the EAE model. We immunized C57BL/6 mice with myelin oligodendrocyte glycoprotein (MOG) 35–55 peptide and treated them with either a vehicle, DMF, bone marrow–derived tolDCs loaded with MOG_35–55_, or the combination of DMF and peptide-loaded bone marrow–derived tolDCs. DMF + tolDCs treatment of EAE mice induced a significant reduction in the clinical score, in comparison with either DMF or tolDC monotherapies, which had a comparable effect (Figure 10A). In addition, we isolated and analyzed CD4^+^ T cell infiltrates in mouse spinal cords. Mice treated with the combined therapy showed a reduced infiltration of pathogenic IL-17–producing CD4^+^ T cells in comparison with monotherapies (Figure 10B). We then analyzed the percentage of total CD4^+^ FoxP3^+^ CD25^+^ Tregs present in mouse spleens. However, statistical significance was not reached in any comparison (Figure 10C). Finally, to evaluate whether any therapy was able to induce hyporesponsiveness against the immunizing antigen, we stimulated EAE-derived spleens with MOG_35–55_ peptide for 4 days and checked for splenocyte proliferation. Strikingly, we observed a reduction in MOG splenocyte reactivity in the combined therapy group versus vehicle and monotherapies, suggesting a stronger antigen-specific hyporeactivity against the autoantigen MOG (Figure 10D).

## Discussion

Since the FDA approved the first therapeutic cellular products, hundreds of patients have benefited from cell therapies. Indeed, cell therapies open important perspectives on how to treat, and possibly cure, immune-mediated diseases. In this context, antigen-loaded tolDC-based therapies represent a possibility to re-educate the myelin autoreactive immune system of MS patients toward tolerance without causing suppression of physiological immunity. However, tolDCs are autologous therapies generated from patients with different grades of immune dysregulation. Thus, starting material may be carrying a pathogenic and/or inflammatory phenotype imprinted by the environment in which monocytes originated and persisted, eventually leading to DC therapies with suboptimal functionality. This idea is supported by studies addressing the impact of the disease environment on cell therapy starting-material characteristics in T cell immunotherapies for cancer (63–66). However, the same type of studies has not been performed in the context of myeloid regulatory cell therapies, nor autoimmune diseases in general. Our study tested this hypothesis in MS patients in the context of a tolDC-based therapy, revealing an activated, proinflammatory monocyte state in MS versus HDs. MS patients had reduced classical monocytes and increased non-classical and intermediate monocytes. Intermediate monocytes expand in inflammatory conditions (67) and produce high TNF-α (68). Indeed, RNA-Seq data showed upregulation of inflammatory genes (TNF, CCL4) and enrichment of NF-κB, STAT, and Jun pathways. Non-classical monocytes, though occasionally considered antiinflammatory, are often associated with MS and other autoimmune diseases (69). CD45RA^+^ and CD40^+^ markers were enriched in MS monocytes (70, 71), and DNA methylation analysis highlighted inflammatory factors (AP-1, Fos, JunB) related to NF-κB (72). These findings support a proinflammatory CD14^+^ fraction in MS, consistent with dysregulation observed in MS monocytes in other studies (73–77). We hypothesize that inflammation, blood-brain barrier disruption, and elevated proinflammatory cytokines may imprint this phenotype. We cannot exclude the expansion of non-classical and intermediate monocytes as an important factor influencing MS CD14^+^ inflammatory signature. In this sense, single-cell technologies could help identify the exact subpopulation involved in this phenotype. Importantly, MS monocyte proinflammatory signature persists during in vitro differentiation, leading to DCs enriched in inflammatory pathways. mDCs show increased mTOR expression, widespread demethylation, and upregulation of inflammatory factors typical of the NF-κB signature, mimicking changes observed in MS monocytes. This suggests that similar proinflammatory and potentially pathogenic alterations may occur in monocyte-derived DCs in vivo. While several studies highlight alterations in DC subpopulations in MS patients and the EAE model (78–80), few specifically address these changes in monocyte-derived DCs.

Finally, enrichment of NF-κB and mTOR in MS mDCs offers several new targets that could be therapeutically targeted with inhibitors to modulate in vivo immunogenic DCs.

Interestingly, MS tolDCs did not show wide methylation changes as MS mDCs did. This can be explained by previous work from our group (26) highlighting vitamin D_3_ as an epigenetic remodeler. Despite this, MS tolDCs were less able to decrease proliferation in allogeneic MLR experiments in comparison with HD, suggesting that reversion of DNA methylation by itself could not completely reestablish full functionality of MS tolDCs. Indeed, MS tolDCs still show transcriptomic differences, with overexpression of markers linked to DC maturation, activation, and immunogenicity (*CD1c*, *CD1a*, *CD24*) (81–83) and downregulation of *ARG1*, an important factor of the VitD3-tolDC gene program (84), which may partially influence this reduced suppressive capability.

Integration of DNA methylation data revealed that MS monocytes, MS mDCs, and MS tolDCs shared demethylation changes in CpGs related to *AHRR*, the repressor of AHR. Validation of gene expression in MS tolDCs confirmed downregulation of the AHR program, in line with studies highlighting alteration of the AHR pathway in MS (85, 86). At the cellular level, AHR can imprint either pro- or antiinflammatory features in the T cell compartment (57, 87), while its activity in DC is linked to the acquisition of tolerogenic features. Interestingly, a recent study performed on DC-10 cells, a type of monocyte-derived tolDC differentiated in the presence of IL-10, highlighted AHR as a regulator of the DC-10 program through autocrine IL-10 signaling (23). Moreover, this same study showed that MS DC-10 cells are functionally defective in comparison with the ones differentiated from HDs, supporting our findings.

In a context of inflammation, hyperactivation, and dysregulation of tolerogenic mechanisms (88), AHR agonism could overcome MS-intrinsic defects in this signaling pathway, leading to fully functional DCs. Several studies showed that AHR agonism can induce tolDCs in vitro, as well as clinical amelioration in EAE mouse models (51, 89–97). While direct agonism of AHR with FICZ improved tolDC functionality in our data set, real-world administration of this molecule to MS patients is problematic because of its fast pharmacokinetic and low stability (56). In this context, the AHR agonist laquinimod showed promising preclinical results (98, 99), but clinical trials (NCT02284568, NCT01707992) (100, 101) investigating its use in MS patients did not reach their primary endpoints, with higher doses characterized by high toxicity. On the other hand, DMF is already approved as first-line treatment in MS, and its principal mechanism of action involves activation of the transcription factor NRF2 and inhibition of NF-κB (58, 59). Moreover, both direct and indirect interactions between AHR and NRF2 exist, allowing both TFs to induce a tolerogenic signature in DCs through shared targets (60, 61). DMF can also induce antiinflammatory monocytes (102), which are also main drivers of good response to DMF treatment in MS (103). In our study, DMF-treated patients show higher classical monocytes and lower intermediate and non-classical monocytes in comparison with MS patients. Moreover, DMF monocytes expressed lower levels of CD45RA, CD40, and CX3CR1 and higher PD-L1 in comparison with MS patients. Taken together, these results indicate that DMF monocytes appear to have a more regulatory profile in comparison with proinflammatory monocytes encountered in treatment-naive MS patients.

Additionally, DMF and other fumarates reduce costimulatory and maturation markers in DCs, and in vivo DMF can induce IL-10–producing DCs in humans (104–106). We found that DMF synergizes with vitamin D_3_ to produce tolDCs with reduced costimulatory markers, proinflammatory cytokines, and allogeneic proliferation. Except for IL-6 production, DMF’s effects in vitro were similar to those of FICZ. While DMF’s induction of CYP1A1 and possibly of AHR activity was observed, direct interactions between NRF2 and AHR or DMF’s direct action on AHR remain unproven, highlighting the need for further investigation.

Importantly, tolDCs differentiated from patients receiving DMF treatment had comparable functionality to the ones derived from healthy individuals, which had better tolerogenic functionality than the ones produced from naive patients. This effect was also observed by addition of in vitro DMF to MS tolDCs differentiated from naive patients, confirming the involvement of DMF signaling in monocyte-to-tolDC differentiation. Given its ability to inhibit NF-κB signaling and induce an AhR-like functional signature, DMF supplementation in MS patients or addition of DMF ex vivo during differentiation can reverse MS monocytes’ proinflammatory signature, leading to more powerful tolDCs. Addition of DMF to tolDC allogeneic cocultures induced less proliferation and lower production of IFN-γ and IL-1β, suggesting modulation of both T cells and activated myeloid cells. Indeed, both TNF and IL-1β are downstream of NF-κB positive regulation, which is strongly inhibited by DMF. However, an in vivo approach in which DMF is administered to MS patients before and after tolDC generation and administration could offer advantages. Indeed, a DMF plus tolDCs combined therapy ameliorated the clinical score of EAE mice and reduced CD4^+^ Th17 cell CNS infiltration as well as splenocyte reactivity to a myelin antigen, suggesting induction of autoantigen hyporesponsiveness. Thus, we propose a combined therapy approach, in which simultaneous treatment in vivo and in vitro with DMF and tolDCs would exert both synergic and independent effects: on one side the beneficial immunomodulatory effect of DMF, reducing inflammation and imprinting monocytes with a regulatory phenotype, boosting the functionality of tolDCs differentiated from the patient; on the other side in vitro supplementation of DMF to tolDCs during the differentiation, which will further aid the generation of fully potent tolDCs with maximal tolerance induction capability against autoreactive clones in MS and other autoimmune diseases.

## Methods

### Sex as a biological variable.

MS and HD cohorts used for RNA-Seq and DNA methylation analysis were designed to be sex- and age-matched. When comparing HD and MS patients in MLRs, samples were matched by sex and age. Our study examined male and female animals, and similar findings are reported for both sexes.

### Patients and donors.

Whole-blood samples from healthy donors (HDs) and relapsing-remitting MS patients were collected in lithium heparin tubes for RNA-Seq, DNA methylation, qPCR, and flow cytometry experiments comparing MS and HD cells. Only active-phase MS patients, untreated with corticosteroids for 1 month or disease-modifying therapy for 12 months, were included. MS patients on DMF for over 6 months were included for DMF-related experiments. Buffy coats for in vitro validation with FICZ, CH223191, and DMF were obtained from anonymous donors via the Banc de Sang i Teixits (Barcelona, Spain) following World Medical Association Declaration of Helsinki guidelines and informed consent.

### CD14^+^ monocyte isolation.

Protocol for the isolation of CD14^+^ cells from peripheral blood can be found in Supplemental Methods.

### TolDC and mDC differentiation.

Extended differentiation protocol of mDCs and tolDCs can be found in Supplemental Methods.

### Flow cytometry analysis of monocytes and DC surface marker expression.

Surface expression of CD11c, CD14, CD83, CD86, CCR7, and HLA-DR protein markers in mDCs and different types of tolDCs from HD or MS patients (without FICZ, CH223191, or DMF) was analyzed by flow cytometry. Complete flow cytometry protocol and antibody information can be found in Supplemental Methods.

### Mixed lymphocyte reaction suppression assay.

Information about the mixed lymphocyte reaction suppression assay can be found in Supplemental Methods.

### Mixed lymphocyte reaction T cell polarization assay.

Full information about the mixed lymphocyte reaction T cell polarization assay protocol can be found in Supplemental Methods.

### DNA and RNA extraction.

DNA and RNA extraction protocol can be found in Supplemental Methods.

### Retrotranscription and qPCR.

Retrotranscription and qPCR protocol is reported in Supplemental Methods.

### Bisulfite conversion and DNA methylation analysis.

Detailed protocol for bisulfite conversion and DNA methylation analysis can be found in Supplemental Methods.

### DNA methylation data analysis.

Information on DNA data analysis linked to enrichment of HOMER TF binding motifs, Gene Ontology analysis, and ChromHMM functional state enrichment can be found in Supplemental Methods.

### Bulk RNA-Seq analysis.

Starting from total RNA obtained from either monocytes, mDCs, or different types of tolDCs from HD and MS patients, RNA-Seq libraries were generated and sequenced by Novogene (Cambridge, United Kingdom). Detailed information on sequencing parameters and data analysis pipeline is available in Supplemental Methods.

### Cytokine quantification and metabolic analysis of supernatants.

Complete information about protocols for cytokine quantification and metabolic analysis of supernatants can be found in Supplemental Methods.

### Mice.

Female and male C57BL/6J mice, 8–10 weeks old, were purchased from Envigo Rms Spain SL and housed at the Comparative Medicine and Bioimage Centre of Catalonia (CMCiB) under standard light- and climate-controlled conditions, with standard chow diet and water provided ad libitum.

### Bone marrow–derived DC differentiation.

Bone marrow–derived DC differentiation protocol can be found in Supplemental Methods.

### Induction of EAE, clinical follow-up, and in vivo treatment of EAE mice.

A detailed protocol for the induction of the EAE model, clinical follow-up, and DMF and tolDC administration can be found in Supplemental Methods.

### Infiltrating lymphocyte analysis.

Detailed infiltrating lymphocyte analysis can be found in Supplemental Methods.

### Analysis of Tregs in mouse splenocytes.

Detailed protocol for analysis of spleen CD4^+^ FoxP3^+^ Tregs can be found in Supplemental Methods.

### Antigen-specific splenocyte reactivity.

The complete protocol for antigen-specific splenocyte reactivity can be found in Supplemental Methods.

### Statistics.

All statistical analyses were performed using Prism 9.0 software (GraphPad) or R software v4.3.1, with either parametric or non-parametric tests depending on the normality of the data set. Exact statistical tests are reported in figure captions. Results are shown in plots as mean ± SD, unless noted differently and with exact *P* values.

### Study approval.

This study was approved by the Germans Trias i Pujol Hospital ethics committee, with written informed consent obtained from all patients and healthy donors. Mouse experiments were approved by the Comparative Medicine and Bioimage Centre of Catalonia (CMCiB) Ethics Committee and the Government of Catalonia. Anonymous blood samples for experiments involving buffy coats were obtained through the Banc de Sang i Teixits (Barcelona, Spain) following institutional standard operating procedures for blood donation in accordance with the World Medical Association Declaration of Helsinki, including signed informed consent.

### Data and code availability.

Raw data are in the Supporting Data Values file. DNA methylation and RNA-Seq data are available in the NCBI’s Gene Expression Omnibus database under accession numbers GSE267660 and GSE267576. No original code is reported. Further details for data reanalysis are available upon request.

## Author contributions

EMC, EB, and FF conceived and designed the study. FF, JW, RDG, MJM, and AAM performed the experiments. FF, GGT, and AGFB performed the bioinformatics analyses. FF, EB, EMC, and JW analyzed results. EMC and EB supervised the study. SPR and CRT provided clinical samples. All authors participated in discussions and interpreted the results. FF, EB, and EMC wrote the manuscript.

## Supplementary Material

Supplemental data

Supplemental table 1

Supplemental table 2

Supplemental table 3

Supplemental table 4

Supporting data values

## Version 1 09/17/2024

In-Press Preview

## Version 2 11/01/2024

Electronic publication
