# Supplementary material for: Targeting aryl hydrocarbon receptor functionally restores tolerogenic dendritic cells derived from patients with multiple sclerosis
Source: J Clin Invest. 2024 Nov 1;134(21):e178949. doi: 10.1172/JCI178949 (PMC11527446; doi:10.1172/JCI178949)
Supplement: Supplemental data [file jci-134-178949-s102.pdf]

SUPPLEMENTARY FIGURE LEGENDS

Supplementary Figure 1. (A) & **(B)** Gene ontology (GO) terms associated with CpGs from hypermethylated (a) and hypomethylated (b) clusters as analyzed by GREAT software. Selected categories are shown. Bars represent log-transformed binomial q values of the GO term enrichment for the HD Mono - MS Mono contrast. **c)** Flow cytometry boxplots reporting Median Fluorescent intensity (MFI) of HLA-DR expression in Classical (CD14++ CD16-), Intermediate (CD14+ CD16+) and Non-classical (CD14+ CD16++) monocytes among MS patients and HD, with respect to total monocytes as parent gate (top row), or reporting the % of HLA-DR-positive (second row) monocytes with respect to Classical, Intermediate or Non-Classical subpopulation. *P*-values from Mann-Whitney tests are shown. n=7 per sample group.

Supplementary Figure 2. (A) Gene ontology (GO) terms associated with CpGs from the hypomethylated cluster analyzed with GREAT software. Selected categories are shown. Bars represent log-transformed binomial q values of the GO term enrichment for the HD mDCs - MS mDCs contrast. **(B)** Chromatin functional state enrichment analysis of the differentially hypomethylated probes in HD-MS mDC contrast, based on CD14+ primary cells ChromHMM public data from Roadmap Epigenomics Project. Odds Ratio is reported on a color scale, while the size of the bubble is proportional to LogFDR. Significant enriched categories are shown (FDR < 0.05, odds ratio > 2), including TssA, active TSS; TssAFlnk, flanking active TSS; Tx, strong transcription; TxWk, weak transcription; EnhG, genic enhancers; Enh, enhancers; Het, heterochromatin; TssBiv, bivalent/ poised TSS; BivFlnk, flanking bivalent TSS/Enh; EnhBiv, bivalent enhancer; ReprPC, repressed PolyComb; ReprPCWk, weak repressed PolyComb. **(c)** Box plots showing RNAseq expression count number of *NFKB1* (left) and *TNF* (right) genes in HD mDCs and MS mDCs.

Supplementary Figure 3. Venn diagram showing shared differentially upregulated (a) and downregulated (b) genes across MS Mono, MS mDCs, and MS tolDCs.

Supplementary Figure 4. (A) Box plots of percentage of positive CD4 T cells: Naive , Central Memory, Effector Memory and Terminally Differentiated Effector Memory T cells analyzed through flow cytometry after 6 days of DCs-PBMCs allogeneic cocultures. *P*-values from ANOVA with multiple comparisons are shown (Mixed effects analysis). n=8 per sample group. (**B**) Box plots of percentage of positive total CD4 T cells expressing the activation marker CD38 after 6 days of DCs-PBMCs allogeneic cocultures. n=6 per sample group. P-values from ANOVA with multiple comparisons are shown (Mixed effects analysis). (**C**) Percentage of HLA-DR+ cells inside total CD4 T cell population after 6 days of DC-PBMC cocultures. *P*-values from ANOVA with multiple comparisons (Mixed-effects analysis) are shown in case of statistical significance. (**D**) Proliferation of allogeneic peripheral blood mononuclear cells cocultured with HD mDCs without or with the presence of DMF 5uM or 10 uM (HD mDCs + 5uM or 10uM DMF), in a 1:10 DC-PBMC ratio. Proliferation was assessed as total of VDP450-positive cells. One-way RM ANOVA with multiple comparisons was used to calculate significant differences among groups, reported as *P*-values. n=12 per group.

Supplementary Figure 5. (**A**) Median fluorescence intensity (MFI) of CX3CR1 (first row) or PD-L1 (second row) positive cells with respect to Classical, Intermediate or Non-Classical monocytes. *P*-values obtained from one-way ANOVA with multiple comparisons are shown (Kruskal-Wallis Test with Dunn’s multiple comparisons). PD-L1 MFI for classical monocytes was not detected and thus was not shown. n=7 or n=4 depending on the sample group.

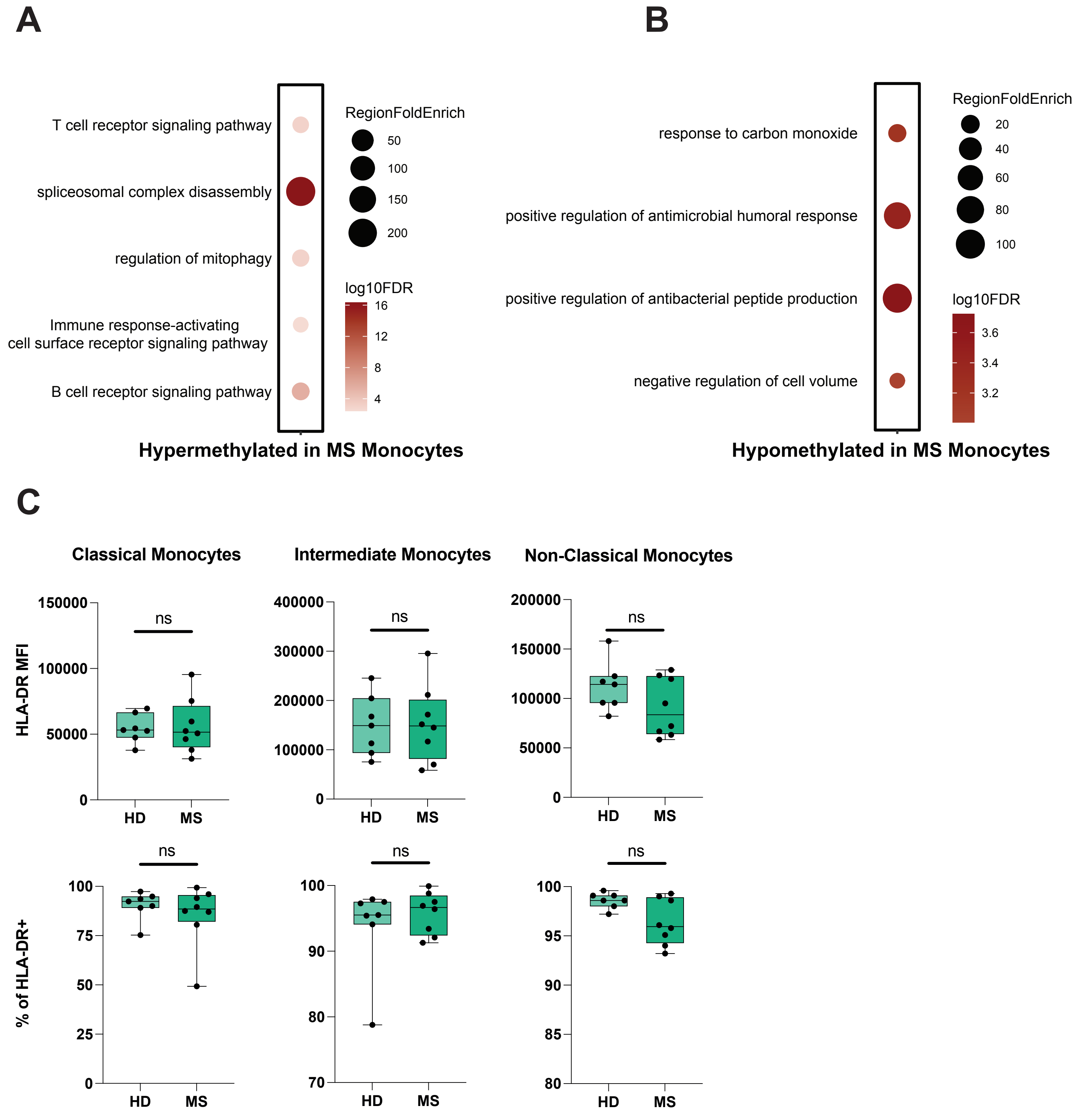
A
T cell receptor signaling pathway
spliceosomal complex disassembly
regulation of mitophagy
Immune response-activating cell surface receptor signaling pathway
B cell receptor signaling pathway
RegionFoldEnrich
50
100
150
200
log10FDR
16
12
8
4
Hypermethylated in MS Monocytes
B
response to carbon monoxide
positive regulation of antimicrobial humoral response
positive regulation of antibacterial peptide production
negative regulation of cell volume
RegionFoldEnrich
20
40
60
80
100
log10FDR
3.6
3.4
3.2
Hypomethylated in MS Monocytes
C
Classical Monocytes
Intermediate Monocytes
Non-Classical Monocytes
HLA-DR MFI
% of HLA-DR+
ns
HD
MS

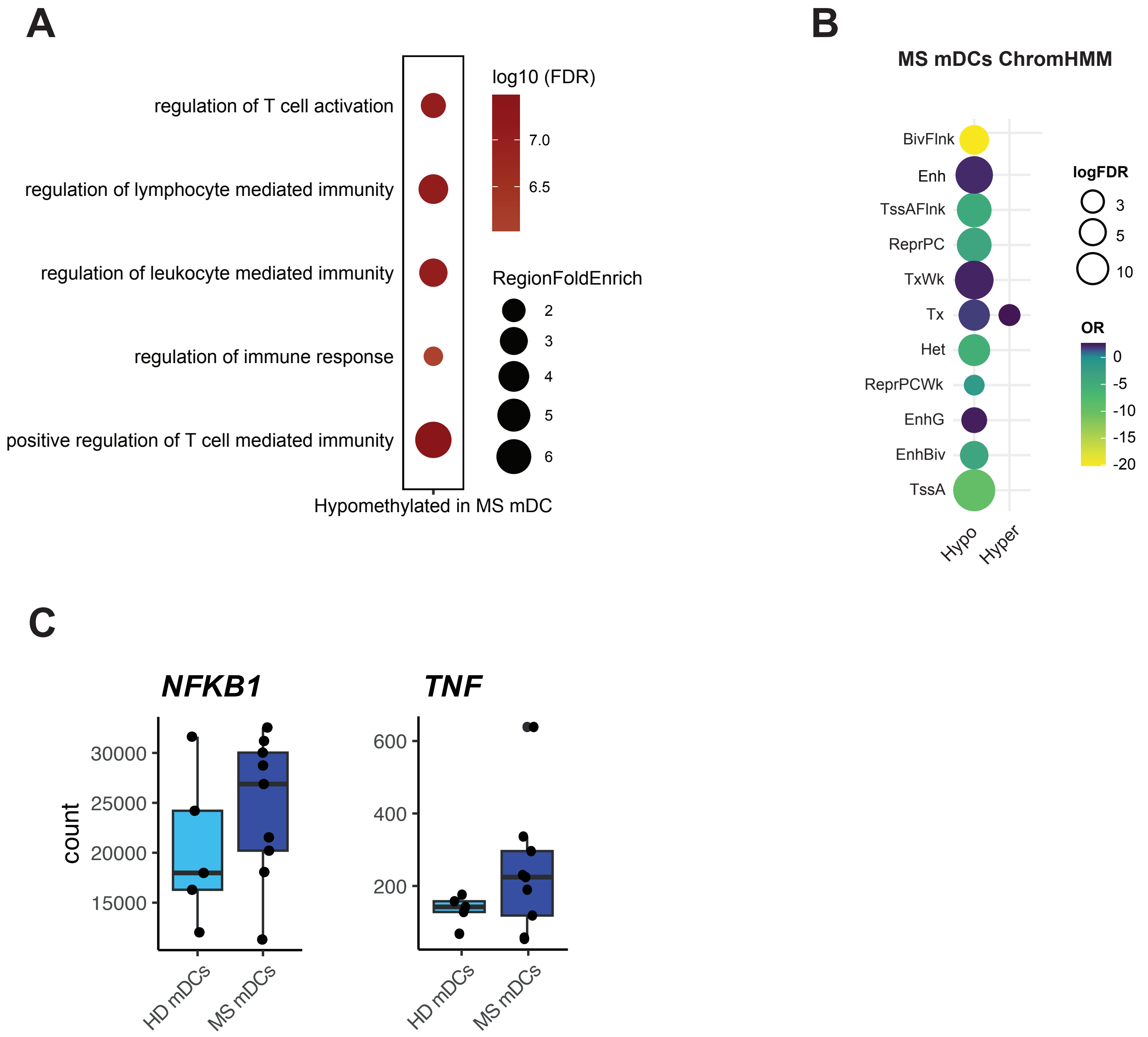
A
regulation of T cell activation
regulation of lymphocyte mediated immunity
regulation of leukocyte mediated immunity
regulation of immune response
positive regulation of T cell mediated immunity
Hypomethylated in MS mDC
log10 (FDR)
7.0
6.5
RegionFoldEnrich
2
3
4
5
6
B
MS mDCs ChromHMM
BivFlnk
Enh
TssAFlnk
ReprPC
TxWk
Tx
Het
ReprPCWk
EnhG
EnhBiv
TssA
Hypo
Hyper
logFDR
3
5
10
OR
0
-5
-10
-15
-20
C
NFKB1
TNF
count
30000
25000
20000
15000
600
400
200
HD mDCs
MS mDCs
HD mDCs
MS mDCs

A

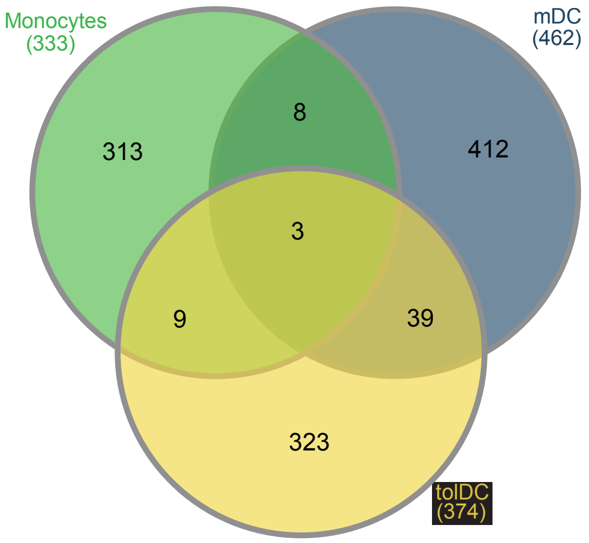
Monocytes
(333)
mDC
(462)
313
8
412
3
9
39
323
tolDC
(374)

B

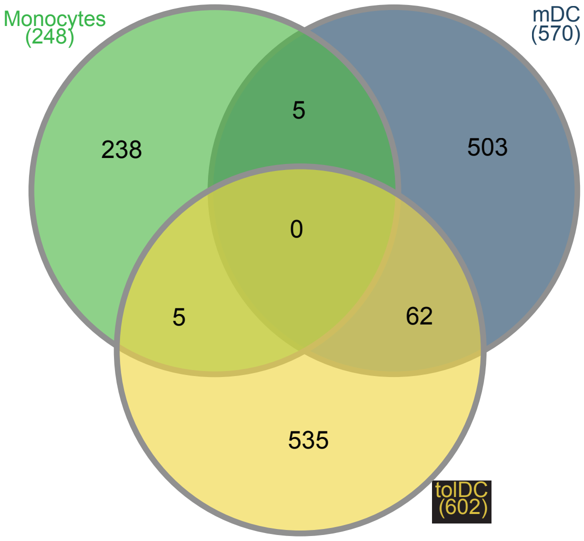
Monocytes
(248)
mDC
(570)
5
238
503
0
5
62
535
tolDC
(602)

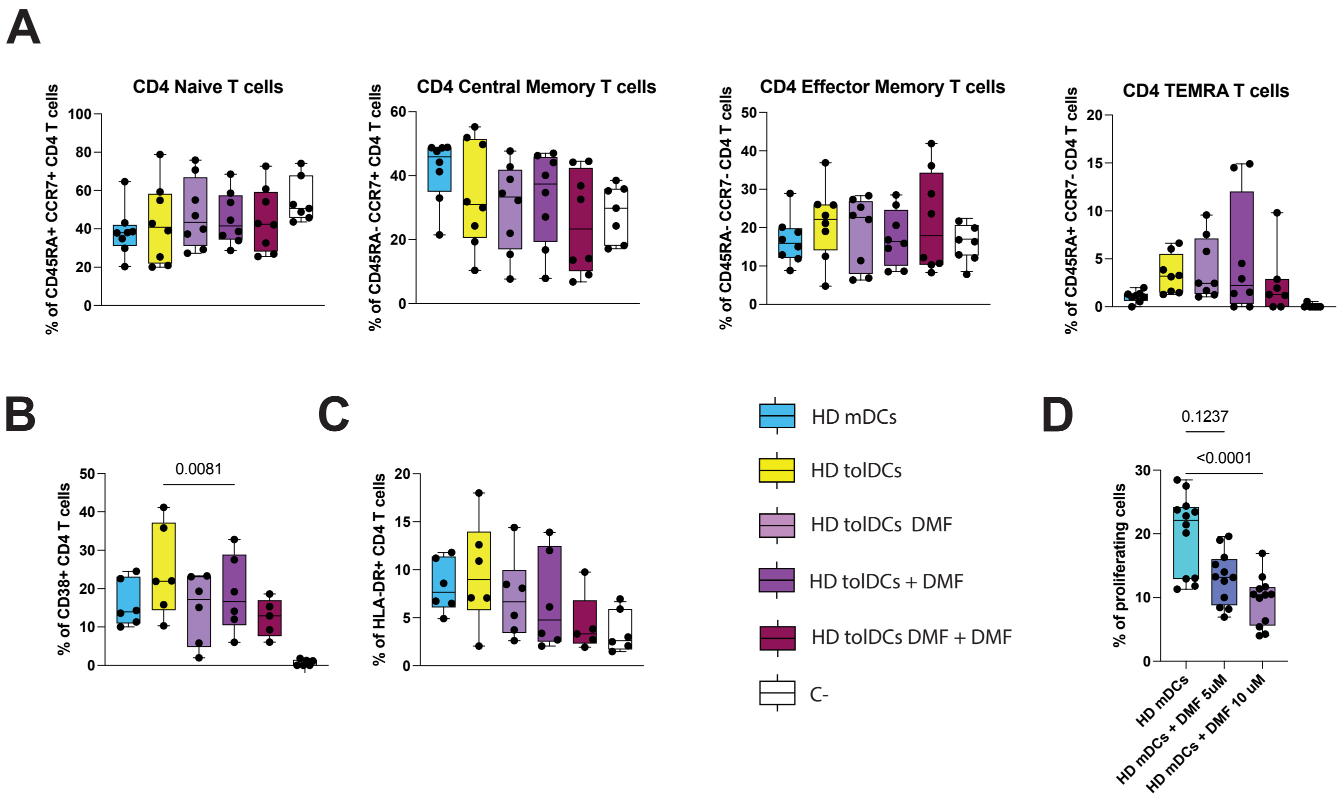

A
CD4 Naive T cells
% of CD45RA+ CCR7+ CD4 T cells
CD4 Central Memory T cells
% of CD45RA- CCR7+ CD4 T cells
CD4 Effector Memory T cells
% of CD45RA- CCR7- CD4 T cells
CD4 TEMRA T cells
% of CD45RA+ CCR7- CD4 T cells
B
% of CD38+ CD4 T cells
0.0081
C
% of HLA-DR+ CD4 T cells
HD mDCs
HD tolDCs
HD tolDCs DMF
HD tolDCs + DMF
HD tolDCs DMF + DMF
C-
D
% of proliferating cells
0.1237
<0.0001
HD mDCs
HD mDCs + DMF 5uM
HD mDCs + DMF 10 uM

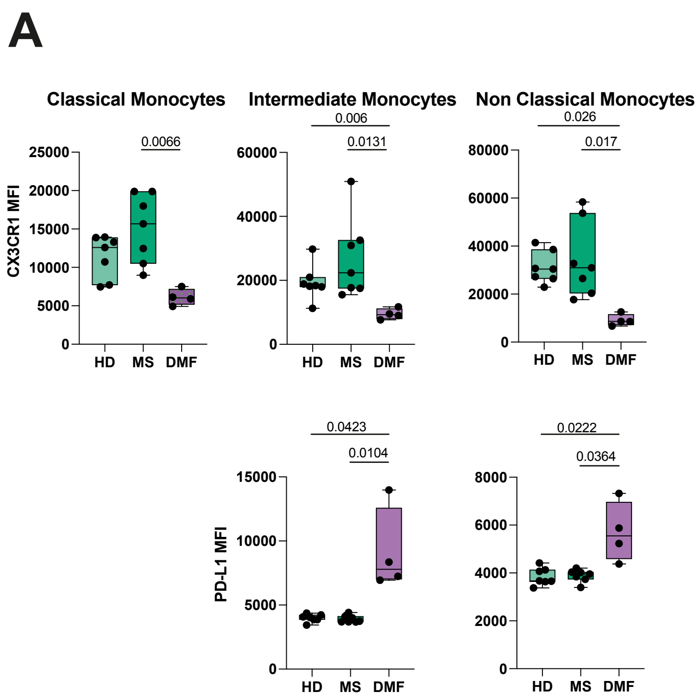
A
Classical Monocytes
Intermediate Monocytes
Non Classical Monocytes
CX3CR1 MFI
PD-L1 MFI
HD
MS
DMF
0.0066
0.006
0.0131
0.026
0.017
0.0423
0.0104
0.0222
0.0364

CD14+ Monocyte isolation

MS patient and HD whole blood and buffy coat samples were processed by firstly enriching the CD14+ fraction using the RosetteSep® Human Monocyte Enrichment Kit (StemCell Technologies, Vancouver, Canada) prior to a density gradient separation using ficoll-hypaque (Rafer, Zaragoza, Spain). Then, positive selection of CD14+ cells was performed using the EasySep® Human CD14 Positive Selection Kit (StemCell), following the manufacturer's instructions. Cell viability was determined using 7-amino-actinomycin D (7-AAD) (BD Biosciences, Cat:51-68981, Franklin Lakes, NK, USA) and phycoerythrin (PE)-conjugated annexin V (Immunotools, Cat:31490014, Friesoythe, Germany) staining for 20 min at 4°C, protected from light, and cell counts were quantified simultaneously using PerfectCount beads (Cytognos, Salamanca, Spain) and Trypan Blue staining (Gibco™). Samples were analyzed on a FACSCanto II flow cytometer (BD Biosciences), and monocyte purity was determined using forward and side scatter gating strategies on FACSDiva software (BD Biosciences). If monocyte purity was > 90%, the isolated CD14+ fraction was used for downstream applications (RNAseq, DNA methylation arrays) and/or to differentiate mDCs or tolDCs.

TolDC and mDC differentiation

In order to differentiated DC, CD14+ cells were cultured for 6 days in 24-well plates at 37°C at a density of 1 × 106 cells/ml in IMDM culture medium (Gibco™, Thermo Fisher Scientific), together with the addition of 400 U/ml granulocyte-macrophage colony-stimulating factor (GM-CSF) and 500 U/ml IL-4 (both from Peprotech, London, UK), 2% L-Glutamine (Sigma-Aldrich), 2% Human serum (Sigma-Aldrich)

and 25 mM HEPES (Gibco™, Thermo Fisher Scientific). The whole medium and cytokines were replenished on day 4. For the generation of mDC, on day 4 a maturation cocktail comprehending 1,000 U/ml IL-1β (Peprotech), 1,000 U/ml TNF-α (Peprotech) and 1 μM prostaglandin E2 (Pfizer, New York, USA) was added. In case of tolDC differentiation, in addition to the maturation cocktail, we added 1 nM vitamin D3 (Calcijex, Abbott, Chicago, IL, USA) on days 0 and 4 for the differentiation of vitD3-tolDC. In case of experiments involving AhR modulation, 18 μM 6-Formylindolo[3,2-b] carbazole (FICZ) AhR Agonist (Invivogen) or 30 μM 2-methyl-2H-pyrazole-3-carboxylic acid (CH223191) AhR Antagonist (Invivogen) were added onday 0 and day 4 along the differentiation. In experiments involving DMF, 10 μM dimethyl fumarate (Sigma) wad added on day 0 and day 4 along the differentiation. Finally, on day 6, cells were harvested, washed twice and used for downstream applications after viability quality control using 7-amino-actinomycin D (7-AAD) (BD Biosciences, Franklin Lakes, NK, USA) and phycoerythrin (PE)-conjugated annexin V (Immunotools, Friesoythe, Germany).

Flow Cytometry analysis of monocytes and DC surface marker expression

Cell suspensions were incubated for 20 min, protected from light, with the appropriate amounts of monoclonal antibodies anti-CD11c PE-Cy7 (BD Biosciences, ot:2124742 Cat:561356 Clone: B-ly6), CD14 V450 (BD Biosciences, ot:2122409 Cat: 560349 Clone: MφP9), CD83 APC (Biolegend, Lot:3062755 Cat: 551073), CD86 FITC (Biolegend, Lot:1309414 Cat: 555657), CCR7 PE (Biolegend, Lot: B375060 Cat:353204 Clone: G043H7) and HLA-DR V500 (BD Biosciences, Lot: 3156363 Cat: 561224 Clone: G46-6). Subsequently, at least 5000 CD11c+ cells for each cell condition were acquired using a FACSCanto II flow cytometer and analyzed using

FACSDiva software. Analysis of percentages of monocytes subsets in HD, MS and MS DMF patients were performed incubating for 30 minutes 3 million peripheral blood mononuclear cells with anti-CD14 Spark Blue™ 550 (BioLegend, cat: 367147, clone 63D3), CD16 PE-AF700 (ThermoFisher, clone: 3G8), anti-CX3CR1 Brilliant Violet 711™ (BioLegend, clone 2A9-1), anti-PD-L1 Brilliant Violet 785™ (BioLegend, 29E.2A3) antibodies and acquiring the samples on a Cytek Aurora Spectral Flower cytometer and analyzed in OMIQ software.

DNA and RNA extraction

DNA from monocytes, mDC and different types of tolDC from HD and MS patients was extracted with a DNeasy Blood & Tissue Kits (Qiagen) following manufacturer's instructions, while total RNA was isolated by using RNeasy Blood & Tissue Kits kit (Qiagen) following manufacturer's instructions. DNA and RNA was quantified with a Nanodrop ND-1000 spectrophotometer (ThermoFisher Scientific) and used for downstream applications.

Retrotranscription and qPCR

Total RNA was retrotranscribed into cDNA by using the High-Capacity cDNA Reverse Transcription Kit (Applied Biosystems, Foster City, CA, USA). cDNA was then used to perform a quantitative RT-qPCR reaction prepared with LightCycler480 SYBR Green I Master (Roche) and analyzed with a LightCycler 480 instrument (Roche). Primers used in this analysis were designed with Primer3 software (1) or bought from commercial vendors (ThermoFisher Scientific). B2M was used as a housekeeping gene and ΔΔCt method was used to analyze the relative quantities of genes of interest.

Bone Marrow-Derived Dendritic Cell Differentiation

BMDC were developed as previously described, obtaining progenitor bone marrow cells from the femurs and tibiae from C57BL/6 donor mice and culturing them in RPMI medium supplemented with 2% L-Glutamine (Sigma-Aldrich), 10% FBS, 1% Sodium pyruvate (Sigma-Aldrich), and 1% penicillin-streptomycin (Thermo-Fisher Scientific) in the presence of 1000 IU/mL of murine granulocyte-macrophage colony-stimulating factor (GM-CSF) (Peprotech). For the generation of VitD3-tolDC, 500IU/mL Calcitriol (Kern Pharma) was added for 8 days. On day 7, 0.1 mg/mL of lipopolysaccharide (LPS) (Sigma-Aldrich) was added to the culture medium of mDCs and VitD3-tolDCs for 22-24h. Next, 10 µM myelin oligodendrocyte glycoprotein 35-55 (MOG35-55) peptide was added for 18 hours to pulse the DC. Ultimately, the VitD3-tolDC-MOG cells were cryopreserved in batches of 10^7 cells and stored in liquid nitrogen until required. The VitD3-tolDC-MOG were characterised by assessing their phenotype and functionality (mixed allogeneic lymphocyte reaction suppression assay).

Infiltrating Lymphocyte analysis

Spinal cords were collected on day post injection 24 by flushing them from the spinal column with PBS. After mechanical disaggregation and enzymatic digestion with 1mg/ml DNase I (11284932001, Roche) and 1 mg/ml collagenase A (C2674, Sigma-Aldrich) for 30 min at 37°C, myelin debris was removed using a Percoll gradient centrifugation. The cells were resuspended with 30% Percoll, added onto a 70% Percoll solution, and centrifuged at 500 x g for 20 min at room temperature. Cells were collected at the interface. Next, for the intracellular staining of infiltrating

lymphocytes, the cells were stimulated with 25 ng/mL PMA (P8139, SigmaAldrich) and 1 µg/mL ionomycin (I3909, Sigma-Aldrich) for 4h. During the last 2h of incubation the 100 ug/ml brefeldin A (B5936, Sigma-Aldrich) was added. Cells were then blocked with anti-mouse CD16/32 (14-0161-85, Invitrogen) and dead cells were labeled with Fixable Viability Stain 575V (565694, BD Biosciences). We then performed surface staining with antibodies against CD3 (100306, Biolegend) and CD4 (46–0042, eBiosciences 565650, BD Pharmigen). Cells were fixated for 15 min with 2% PFA and kept in FacsFlow overnight. Intracellular staining of cytokines was performed to detect IL-17A (563354, BD Horizon), IFNγ (554413, BD Pharmingen), IL-4 (560699, BD Pharmingen), and IL-10 (554467, BD Pharmingen). First, cells were permeabilized with Permeabilization Buffer (00-8333-56, Invitrogen) and incubated for 30 min at 4°C with the antibody mix. Samples were acquired in a BD LSRFortessa flow cytometer and data was analyzed with the OMIQ software.

Analysis of regulatory T cells in mouse splenocytes

Suspension of murine splenocytes was obtained by grinding the spleens through a 70µm nylon cell strainer at dpi 24. Cells were labeled with Fixable Viability Stain 575V. To analyze Treg cell population in the spleen, antibodies against CD3 (100306, Biolegend), CD4 (560468, BD Horizon), CD25 (558642, BD Pharmigen), and Foxp3 (560401, BD Pharmigen) were used. After staining the surface markers, the manufacturer's instructions were followed for the intracellular labeling of Foxp3 using the eBioscience™ Foxp3/Transcription Factor Staining Buffer Set (00-5523-00, Invitrogen). Samples were acquired in a BD LSRFortessa flow cytometer and data was analyzed with the OMIQ software.

Induction of EAE, Clinical Follow-Up and In Vivo Treatment of EAE Mice

EAE was induced by immunizing the mice subcutaneously with 100 µg of MOG35–55 (YRSPFSRVVHLYRNGK) (Immunostep, Salamanca, Spain), emulsified in an equal volume (1:1) of Freund's complete adjuvant containing 4 mg/mL Mycobacterium tuberculosis (strain H37RA, Difco, Detroit, MI, USA). Furthermore, mice were treated intravenously with 250 ng pertussis toxin (Sigma Chemical, St. Louis, MO, USA) on day 0 and day 2 post-immunization (dpi). Animals were weighed and examined daily for welfare and clinical signs. The following criteria were used for clinical evaluation: 0, asymptomatic; 0.5, loss of distal half of tail tone; 1, loss of entire tail tone; 1.5, hind limb weakness; 2, hind limb paralysis; 2.5, hind limb paraplegia; 3, forelimb weakness; 4, quadriparesis; 4.5, severe quadriparesis; 5, quadriplegia; and 6, death. Endpoint criteria were established to minimize suffering and ensure animal welfare. For the DMF treatment, mice were treated daily with vehicle 1 (methylcellulose) or DMF (100 mg/kg body weight, Sigma-Aldrich) suspended in 0,8% methylcellulose (Sigma-Adrich). DMF or vehicle 1 was administered through oral gavage from dpi 3 until the end of the experiment. Regarding the VitD3-tolDC treatment, vehicle 2 (PBS) or 1x10^6 VitD3-tolDC-MOG was administered intravenously on dpi 13, 17, 21 and 24. In order to compare the combined therapy with both monotherapies, the different groups of treatment were: vehicle 1 and vehicle 2, DMF and vehicle 2, VitD3-tolDC-MOG and vehicle 1, and DMF and VitD3-tolDC-MOG.

Antigen-Specific T Cell Reactivity

To study antigen-specific reactivity, splenocytes from all treatment groups were cultured in a 96-well plate at 1,5 × 10^5 cells/well in 200 µL of IMDM (supplemented

with 10% FBS, 2% L-Glutamine, 1% penicillin/streptomycin and 1% sodium pyruvate) containing either 5 μM MOG35-55, 5 μM phytohemagglutinin (PHA) (Sigma-Aldrich) (positive control) or culture medium (negative control). After 72 h of culture, 1 μCi/well of [3H]-thymidine (PerkinElmer) was added for the last 18 h of culture. The stimulation index (SI) for each stimulus was calculated as the mean counts per minute (cpm) of antigen-stimulated cultures divided by the mean cpm of the non-stimulated cultures.

Mixed Lymphocyte Reaction Suppression Assay

To isolate allogeneic PBMC, whole blood samples of healthy individuals were processed by ficoll-hypaque density gradient separation. Then, PBCMs were stained with BD Horizon™ Violet Proliferation Dye 450 (BD Bioscience) DNA dye and co-cultured in 96-well round bottom plates, in a total volume of 200 μl of supplemented RPMI medium, at a ratio of 1:20 with either MS-derived or HD-derived mDC, tolDC, tolDC FICZ, tolDC CH or tolDC DMF, according to the experiment. Cells were kept for 4 days at 37°C in a 5% CO2 atmosphere, and then the V450 positive fraction was calculated for each condition by using a FACS BD Lyrics flow cytometer. Negative controls comprehending PBMCs-only and positive controls with 50 ng/mL phorbol 12-myristate-13-acetate (PMA) and 500 ng/mL ionomycin (Thermo Fisher Scientific) were also used. Percentages of cell proliferating in the different tolDC conditions were then normalized to the percentage of proliferation induced by mDC, used as controls, and multiplied by 100, obtaining the percentage a suppression of proliferation.

Mixed Lymphocyte Reaction T cell Polarization assay

PBMCs were isolated from sex-matched healthy donors' buffy coats as for MLR Suppression assays. Then, PBCMs were co-cultured in 96-well round bottom plates, in a total volume of 200 µl of supplemented RPMI medium, at a ratio of 1:2 with different types of HD DCs (HD mDCs, HD tolDCs, HD tolDCs + DMF, HD tolDCs DMF or HD tolDCs DMF + DMF) and in presence/absence of 10 µM DMF during the differentiation to tolDCs or during the coculture. Negative controls comprehending PBMCs-only were also used. Cells were kept for 6 days at 37°C in a 5% CO2 atmosphere, stained with a panel of antibodies comprehending anti-CD3 V450 (BD Biosciences, Cat:560365 Clone: UCHT1), anti-CD4 PerCPCy5.5 (BD Biosciences, Cat:560650 Clone: RPA-T4), anti-CD45RA Pe-Cy7 (BD Biosciences, Cat:560675 Clone: HI100), anti-CCR7 PE (Biolegend, Cat:353204 Clone: G043H7), anti-HLA-DR (Biolegend, Cat: 561224 Clone: G46-6), anti-CD38 (Biolegend, Cat: 555462) anti-CCR6 and v-CXCR3 AF488 (Biolegend, Cat:353710 Clone: G025H7) and then acquired in a LSR Fortessa flow cytometer (BD Biosciences).

Bisulfite conversion and DNA methylation analysis

500 ng of genomic DNA was BS-converted with an EZ DNA Methylation-Gold kit (Zymo Research), following manufacturer's instructions and were hybridized in Infinium MethylationEPIC BeadChip arrays (Illumina, Inc., San Diego, CA, USA) following the manufacturer's instructions. This technology permits to analyze more than 850,000 methylation sites per sample at the level of single nucleotides, covering 99% of the reference sequence (RefSeq) genes and 95% of CpG islands. Image processing and intensity data extraction procedures have been performed as previously described (2). In principle, each methylation data point was constituted

from the combination of fluorescent intensities of Cy3 and Cy5 from the methylated and unmethylated alleles. Background intensity was calculated from a set of negative controls and subtracted from each data point. Data points were then analyzed by using beta (β) values and M values. β values correspond to the ratio among the methylated probe intensity to the overall intensity, given by the sum of the methylated and unmethylated probe intensities. M values are calculated as the log2 ratio of the intensities of the methylated versus unmethylated probes. Raw methylation data were preprocessed with the minfi package (3) and data quality was evaluated by using the minfi and RnBeads packages (4,5). After Snoob normalization, we used M values to obtain adjusted p-values (Benjamini-Hochberg-calculated FDR) between sample groups by using a eBayes-moderated paired t-test using the limma R package (6). Many different criteria have been proposed in order to represent differentially methylated CpG among experimental conditions. In this study, a probe was considered differentially methylated if presenting a false discovery rate smaller than 0.05 ($FDR < 0.05$) and a β value greater than 5% ($\beta > 5\%$). Hierarchical clustering using Pearson correlation distances and average linkage criteria and DMP heatmaps were realized by using functions from the gplots and ComplexHeatmap R packages.

DNA Methylation Data Analysis

To assess enrichment of transcription factor motifs in our DNA methylation dataset we used the HOMER software (7). Specifically, we used the findMotifsGenome.pl algorithm (with settings -size 250 -cpg) to individuate significant enrichment against a

background sequence adjusted to have similar CpG and GC contents. Genomic regions were annotated with the annotatePeaks.pl algorithm. To assess the position relative to a CpG island, we used ‘hg19_cpgs’ annotation in the annotatr R package. GREAT software (8) was used to obtain gene ontologies by using the single nearest gene option to define associations between genomic regions and coding genes. Chromatin functional state enrichment of DMPs was analysed using as background public available CD14 primary cells data obtained from the NIH Roadmap Epigenomics Project (http://www.roadmapepigenomics.org) generated with the ChromHMM software (9) by using a 15-state model — primary HMM — constructed with data from 5 histone modification marks and checking for enrichment and significance by Fisher’s exact tests.

Bulk RNAseq Analysis

Samples were sequenced in 150-bp paired-end using an Illumina NovaSeq 6000 machine and at least 40 million reads were obtained for each sample. Fastq files were aligned to the hg38 transcriptome using HISAT2 (10) and reads mapped in proper pairs and primary alignments were selected with SAMtools (11). Then, reads were assigned to genes with featureCounts (12) and differentially expressed genes (DEGs) were calculated with DESeq2 (13). The Normal shrinkage algorithm was used and genes with an FDR < 0.05 and a LogFC $> \pm 0.5$ were considered DEGs. Inference of transcription factors activity from gene expression values were assessed using DoRothEA (14).

Cytokine Quantification of culture supernatants and Metabolic Analysis of supernatants

The production of IL-6, IL-12p70, IL-1β was quantified simultaneously at day 6 of differentiation in the culture supernatants of tolDC, tolDC FICZ and tolDC DMF by using LEGENDplexTM Human Essential Immune Response Panel according to manufacturer's instructions. Samples were acquired on an LSR Fortessa flow cytometer (BD Biosciences) and analyzed using FACSDiva software. Quantification of IFNγ, TNFα and IL-1β in allogeneic MLRs supernatants was performed with the same technique at day 6 of co-culture. Glucose consumption, secretion of lactate and pH quantification were performed on supernatants collected on day 6 of differentiation to tolDC, tolDC + FICZ or tolDC + CH223191. Glucose and lactate concentrations were determined in an AU5800 platform (Bekman Coulter; Clare, Ireland) using a standard hexokinase method and a lactate oxidase reaction, respectively. For pH quantification, a direct potentiometry method was used in a Gem Premier 4000 analyzer (Werfen, MA, USA).

Supplementary Methods Bibliography

1. Koressaar T, Remm M. Enhancements and modifications of primer design program Primer3. *Bioinformatics*. 2007;23(10):1289-1291. doi:10.1093/bioinformatics/btm091

2. Bibikova M, Lin Z, Zhou L, et al. High-throughput DNA methylation profiling using universal bead arrays. *Genome Res*. 2006;16(3):383-393. doi:10.1101/gr.4410706

3. Aryee MJ, Jaffe AE, Corrada-Bravo H, et al. Minfi: a flexible and comprehensive Bioconductor package for the analysis of Infinium DNA

methylation microarrays. *Bioinformatics*. 2014;30(10):1363-1369. doi:10.1093/bioinformatics/btu049

4. Assenov Y, Müller F, Lutsik P, Walter J, Lengauer T, Bock C. Comprehensive analysis of DNA methylation data with RnBeads. *Nat Methods*. 2014;11(11):1138-1140. doi:10.1038/nmeth.3115

5. Müller F, Scherer M, Assenov Y, et al. RnBeads 2.0: comprehensive analysis of DNA methylation data. *Genome Biol*. 2019;20(1):55. doi:10.1186/s13059-019-1664-9

6. Ritchie ME, Phipson B, Wu D, et al. limma powers differential expression analyses for RNA-sequencing and microarray studies. *Nucleic Acids Res*. 2015;43(7):e47. doi:10.1093/nar/gkv007

7. Heinz S, Benner C, Spann N, et al. Simple combinations of lineage-determining transcription factors prime cis-regulatory elements required for macrophage and B cell identities. *Mol Cell*. 2010;38(4):576-589. doi:10.1016/j.molcel.2010.05.004

8. McLean CY, Bristor D, Hiller M, et al. GREAT improves functional interpretation of cis-regulatory regions. *Nat Biotechnol*. 2010;28(5):495-501. doi:10.1038/nbt.1630

9. Ernst J, Kellis M. Chromatin-state discovery and genome annotation with ChromHMM. *Nat Protoc*. 2017;12(12):2478-2492. doi:10.1038/nprot.2017.124

10. Kim D, Paggi JM, Park C, Bennett C, Salzberg SL. Graph-based genome alignment and genotyping with HISAT2 and HISAT-genotype. *Nat Biotechnol*. 2019;37(8):907-915. doi:10.1038/s41587-019-0201-4

11. Li H, Handsaker B, Wysoker A, et al. The Sequence Alignment/Map format and SAMtools. *Bioinformatics*. 2009;25(16):2078-2079. doi:10.1093/bioinformatics/btp352

12. Liao Y, Smyth GK, Shi W. featureCounts: an efficient general purpose program for assigning sequence reads to genomic features. *Bioinformatics*. 2014;30(7):923-930. doi:10.1093/bioinformatics/btt656

13. Love MI, Huber W, Anders S. Moderated estimation of fold change and dispersion for RNA-seq data with DESeq2. *Genome Biol*. 2014;15(12):550. doi:10.1186/s13059-014-0550-8

14. Garcia-Alonso L, Holland CH, Ibrahim MM, Turei D, Saez-Rodriguez J. Benchmark and integration of resources for the estimation of human transcription factor activities. *Genome Res*. 2019;29(8):1363-1375. doi:10.1101/gr.240663.118
